# Dissecting the transcriptome of Parkin (*PRKN*)-linked Parkinson’s disease using 2D and 3D hiPSC-neuronal models

**DOI:** 10.1038/s42003-026-10703-0

**Published:** 2026-07-22

**Authors:** Alessandra Zanon, Emanuela Kerschbamer, Diana A. Riekschnitz, Valentina Gilmozzi, Greta Laurenti, Martin Lang, Anne Grünewald, Philip Seibler, Peter P. Pramstaller, Andrew A. Hicks, Christian X. Weichenberger, Irene Pichler

**Affiliations:** 1 Institute for Biomedicine, Eurac Research, Bolzano, Italy; 2 Department of Clinical Pathology, Hospital of Bolzano, Bolzano, Italy; 3 Infection Immunology Laboratory, Department of Biomedicine, University of Basel, Basel, Switzerland; 4 Luxembourg Centre for Systems Biomedicine (LCSB), University of Luxembourg, Belvaux, Luxembourg; 5 Institute of Neurogenetics, University of Lübeck and University Hospital Schleswig-Holstein, Lübeck, Germany

**Keywords:** Transcriptomics, Mechanisms of disease

## Abstract

Mutations in the *Parkin* (*PRKN*) gene are the most common known cause of autosomal recessive early-onset Parkinson’s disease (PD), and Parkin dysfunction represents a risk factor for idiopathic PD. Parkin is an E3 ubiquitin ligase, involved in protein quality control and the removal of damaged mitochondria. Here, we generate human induced pluripotent stem cell (hiPSC)-derived midbrain dopaminergic neurons using both a 2D differentiation protocol and our recently developed 3D method based on the microencapsulation of hiPSCs in small alginate/fibronectin beads. The transcriptional signature of cells from PD patients carrying *PRKN* mutations is evaluated at the neuronal stage and during the differentiation process. Analyses of mutation-specific gene sets and associated pathways reveal multiple biological processes implicated in *PRKN*-pathology, including synaptic and metabolic function, inflammation, and intracellular trafficking, reflecting a layered disease development. These data map the contribution of Parkin in early PD pathogenesis and progression before overt neurodegeneration.

## Abstract (web summary)

Transcriptomic profiling of PRKN-mutant hiPSCderived dopaminergic neurons in 2D and 3D reveals early alterations in synaptic, metabolic, inflammatory, and trafficking pathways, providing insights into Parkinson’s disease mechanisms.

## Introduction

Parkinson’s disease (PD) is the second most frequent chronic progressive neurodegenerative movement disorder in aging, second only to Alzheimer’s disease (AD)^1^. The progressive degeneration of midbrain dopaminergic (mDA) neurons in the *substantia nigra pars compacta* (*SNpc*) and the presence of alpha-synuclein-positive inclusions in the cytoplasm of neurons, termed Lewy bodies, represent key features of PD pathology. While about 90% of PD cases are classified as idiopathic, in the past two decades, inherited mutations in more than 20 genes have been linked to rare, familial forms of PD and Parkinsonism^2^. Mutations in the *PRKN* gene, encoding Parkin, represent the most common known cause of autosomal recessive early-onset Parkinsonism, accounting for up to 42.2% of cases with an age of onset ≤20 years^3^, and Parkin dysfunction represents a risk factor for idiopathic PD (iPD)^4^. *PRKN* mutations have been identified in numerous families with different genetic backgrounds^5^ and include more than 130 mutations consisting of copy number variations (CNVs) (deletions or multiplications of exons), small deletions/insertions, as well as single nucleotide polymorphisms (SNPs)^6,7^. By studying the effect of *PRKN* mutations, a direct role of mitochondrial dysfunction in the onset of PD has emerged. The Parkin protein is an E3 ubiquitin ligase that has been implicated in several cellular and mitochondrial functions, including the activation of the mitophagy pathway^8,9^, mitochondrial biogenesis^10,11^, mitochondrial DNA (mtDNA) transcription^12^, mitochondrial genome integrity^13^, and the control of the cell’s apoptotic response^14^. Mitochondria are vulnerable to stress induced by gene mutations, aging, and pathogens, leading to the release of damage-associated molecular patterns (DAMPs), including mtDNA, that activate immune signaling pathways. Notably, *PRKN*-mutant neurons showed increased cytosolic mtDNA levels, and *PRKN*-mutant neuron–microglia co-cultures displayed an enhanced inflammatory response following stimulation with lipopolysaccharide (LPS) and mtDNA as compared to controls^15^. Furthermore, during cell stress, Parkin was found to actively inhibit mitochondria-derived vesicle formation and mitochondrial antigen presentation at the cell surface, and in Parkin deficiency, inflammatory conditions trigger mitochondrial antigen presentation in immune cells^16^. Given the complex role of Parkin in determining cell fate in response to mitochondrial damage, it is important to further explore its involvement in cellular processes, directly or indirectly linked to mitochondria, by investigating the effect of disease-causing mutations in physiologically relevant human-derived cellular models. Due to the inaccessibility of viable diseased tissue and the significant loss of mDA neurons in *postmortem* tissue of PD patients^17^, the development of cellular reprogramming technologies provided an innovative alternative by the generation of patient-specific neurons. Human induced pluripotent stem cells (hiPSCs) derived from peripheral cells of PD patients can be differentiated into mDA neurons, providing an unlimited source of neurons for the investigation of cellular mechanisms and deep phenotyping^18,19^.

Here, we generated hiPSC-derived mDA neurons for two PD patients carrying a biallelic mutation in *PRKN* and two control individuals, using both a conventional 2D protocol^18^ and a 3D culture method we recently described based on the microencapsulation and differentiation of hiPSCs in small alginate/fibronectin beads^20^. Targeted expression profiling by RNA-sequencing (RNA-seq) was employed to elucidate the cellular impact of *PRKN* mutations on the molecular events linked to disease development and progression. Our results identify previously unrecognized components of the Parkin-PD network and advance our understanding of the biochemical mechanisms linked to *PRKN*-PD.

## Results

### Time-course RNA-seq analyses reveal the influence of differentiation stage and culture methods on transcriptional profile

**Fig. 1:**
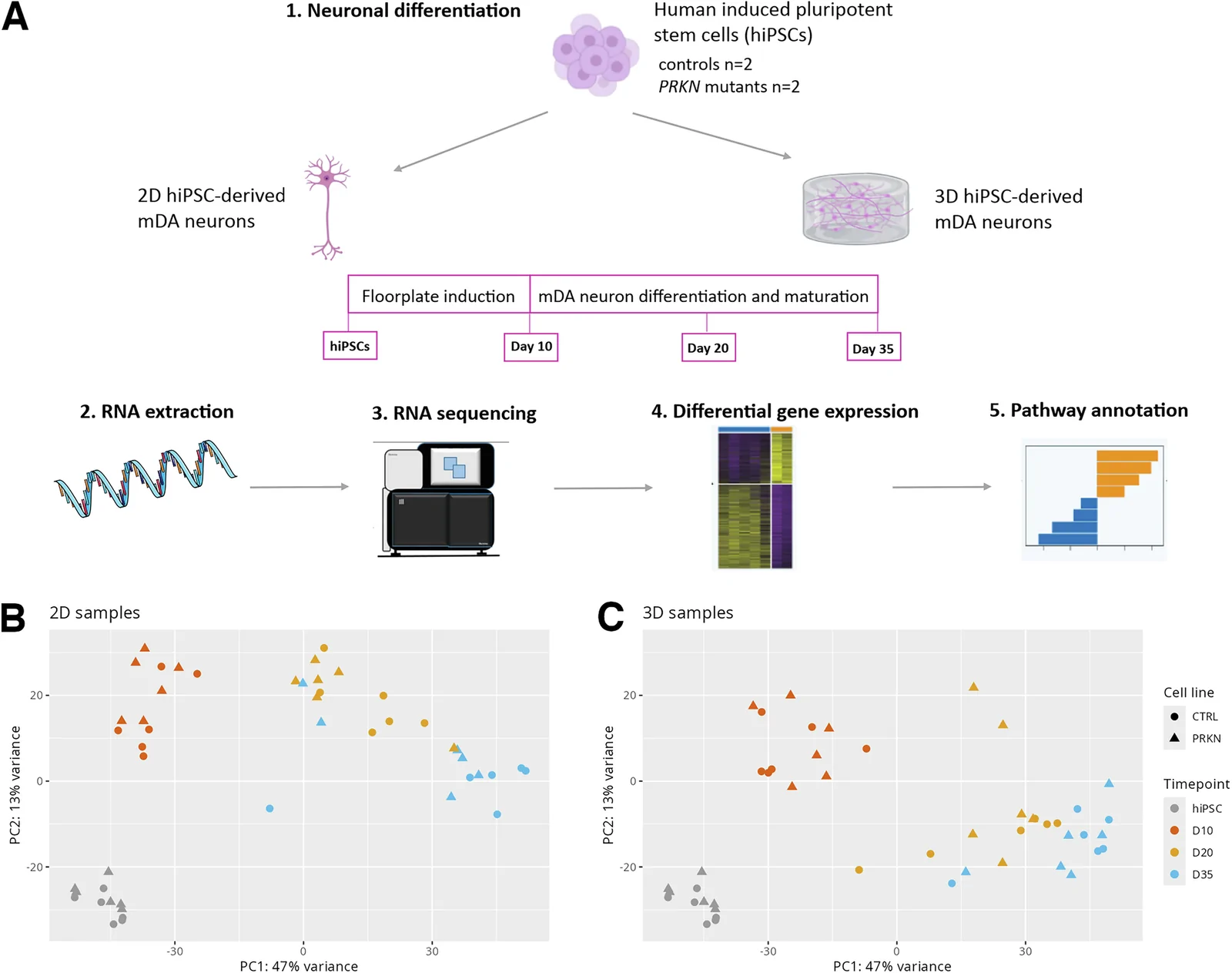
Schematic overview of the transcriptomic workflow and sample distribution of the transcriptomic dataset. **A** RNA-seq analysis using the Illumina NextSeq500 platform was performed at four different timepoints (hiPSC, D10, D20, and D35) during 2D and 3D mDA neuronal differentiation for three independent differentiations of two hiPSC lines carrying PD-associated *PRKN* mutations (homozygous c.971 del A in exon 9 and homozygous exon 3 deletion) and two controls. Figure elements are from BioRender.com. **B** PCA plot showing the *n* = 48 samples included in the 2D differentiations. **C** PCA plot showing the *n* = 48 samples included in the 3D differentiations. Principal component 1 and principal component 2 explained 47% and 13% of the total variance, respectively.

The exact mechanisms by which *PRKN* loss-of-function mutations lead to neurodegeneration are not yet fully understood. Thus, to elucidate molecular signatures associated with the development and progression of PD pathology linked to *PRKN* mutations, we performed an RNA-seq study at four different timepoints (hiPSC, D10, D20, and D35) during mDA neuronal differentiation for two hiPSC lines carrying a homozygous loss-of-function mutation in the *PRKN* gene (*PRKN*-1 and *PRKN*-2) and two control lines (CTRL-1 and CTRL-2) (Supplementary Fig. 1A, Supplementary Table 1). *PRKN*-1 (hiPS-B125) carries a 1 bp-deletion in exon 9 (p.Val324AlafsTer111, rs1562519380) and *PRKN*-2 (hiPS-2011) carries a homozygous deletion of exon 3. Consistent with the predicted loss-of-function effect of both homozygous *PRKN* mutations, Western blot analyses show absent Parkin protein levels in the mutant lines (Supplementary Fig. 1B). mDA neurons were generated by parallel differentiations of patient and control hiPSCs using a conventional 2D protocol^18,21^ and our recently established hydrogel-based 3D protocol^20^. In these cultures, we observed no detectable expression of microglial markers, very low levels of oligodendrocyte precursor and oligodendrocyte lineage markers, low expression of astrocyte markers, and high expression of pan-neuronal and dopaminergic markers at day 35 of differentiation, consistent with the expected cellular composition of hiPSC-derived dopaminergic neuronal cultures (Supplementary Fig. 1C). A comparable neuronal differentiation across all analyzed lines is supported by RNA-seq–based characterization of the expression of the ventral mDA precursor markers *FOXA2* and *LMX1A*, as well as the pan-neuronal and mDA markers *MAP2*, *TUBB3*, *TH*, and *KCNJ6* (*GIRK2*) (Supplementary Fig. 1D). A schematic overview of the transcriptomics study design is given in Fig. 1A.

Out of the 19,000 human protein-coding genes, a total number of 17,801 annotated genes were detected. Principal component analysis (PCA) was performed on the 500 most variably expressed genes to examine overall variability among samples (Fig. 1B, C). The analysis showed differentiation timepoints separated by both PC1 and PC2: hiPSC and D10 samples are clearly grouped together by timepoint and cell line, while D20 and D35 samples are more separated by cell line, both in 2D and 3D samples. This analysis suggested a distinct clustering of samples based on their differentiation stage and genotype.

**Fig. 2:**
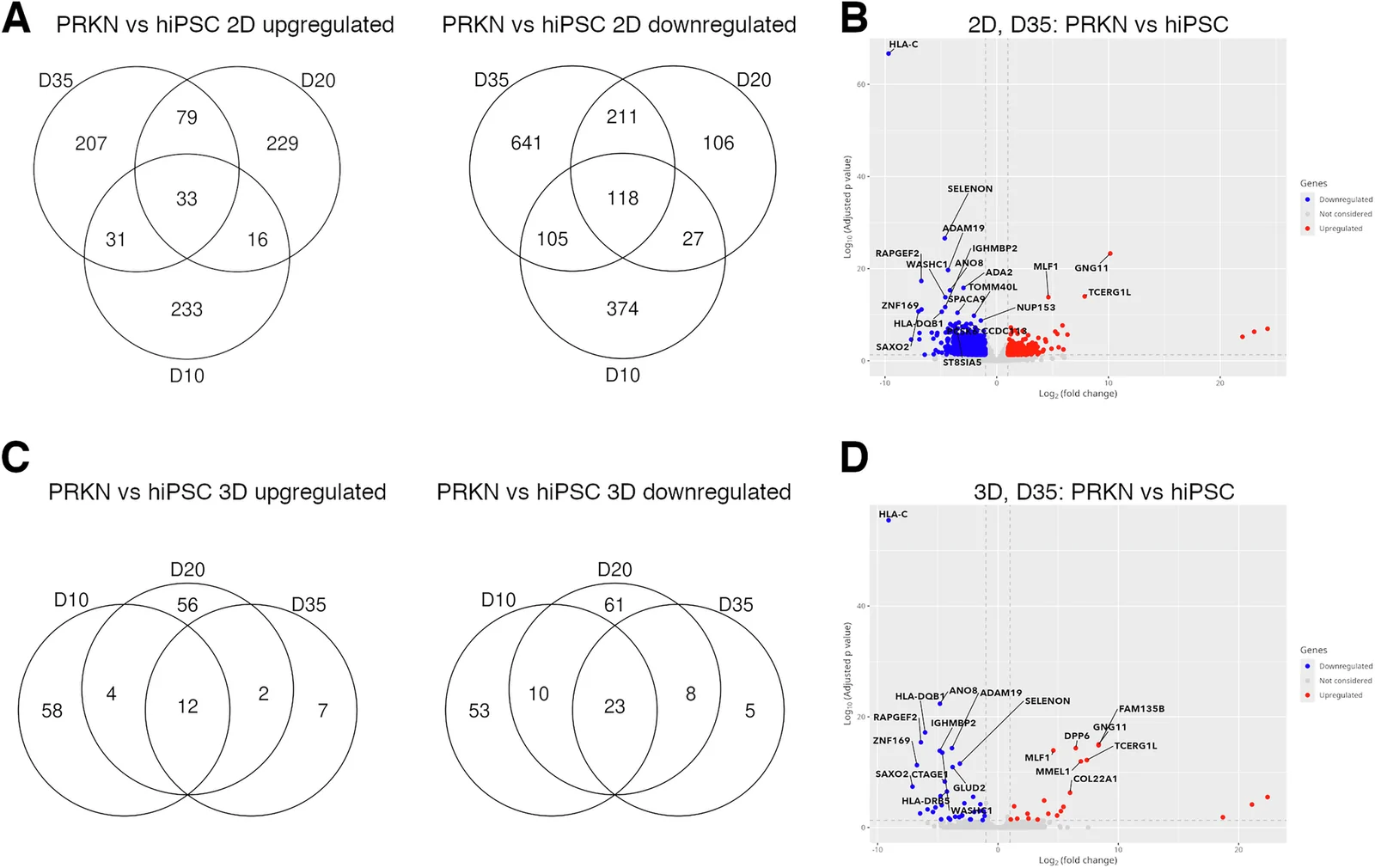
DEGs in *PRKN-*mutant neurons compared to control neurons. **A** Venn diagram showing DEGs, upregulated and downregulated, altered in the same direction across the three timepoints in the 2D culture condition. **B** Volcano plot showing DEGs exclusively altered at D35 of differentiation in the 2D culture condition, highlighting upregulated genes in red and downregulated genes in blue. **C** Venn diagram showing DEGs, upregulated and downregulated, altered in the same direction across the three timepoints in the Alg/Fn 3D culture condition. **D** Volcano plot showing DEGs exclusively altered at D35 of differentiation in the 3D culture system. *n* = 3 independent differentiations.

The analysis of differentially expressed genes in the 2D cultures yielded a total of 151 genes that were significantly and consistently altered in the same direction in *PRKN* mutation carriers compared to controls at all three timepoints (D10, D20, and D35) (33 genes were upregulated, and 118 genes were downregulated) (Fig. 2A and Supplementary Data 1). These DEGs were compiled in a gene list dysregulated by the *PRKN* mutation independently from the neuronal maturation stage. In addition, genes that are differentially expressed exclusively at the single timepoints (D10, *n* = 607; D20, *n* = 335; and D35, *n* = 848) were compiled in a separate gene list, linked to the differentiation stage and potentially to the stage of the pathology (Fig. 2B, Supplementary Fig. 2A, B, and Supplementary Data 1).

In 3D culture, a total of 35 genes (12 upregulated and 23 downregulated genes) were identified in the *PRKN*-mutant cell lines compared to controls at all timepoints (Fig. 2C and Supplementary Data 1). In addition, three lists of the DEGs exclusively altered at the single timepoints (D10, *n* = 111; D20, *n* = 117; D35, *n* = 12) were compiled (Fig. 2D, Supplementary Fig. 2C, D, and Supplementary Data 1).

### In vitro differentiation of hiPSC-derived mDA neurons mimics the in vivo process

**Fig. 3:**
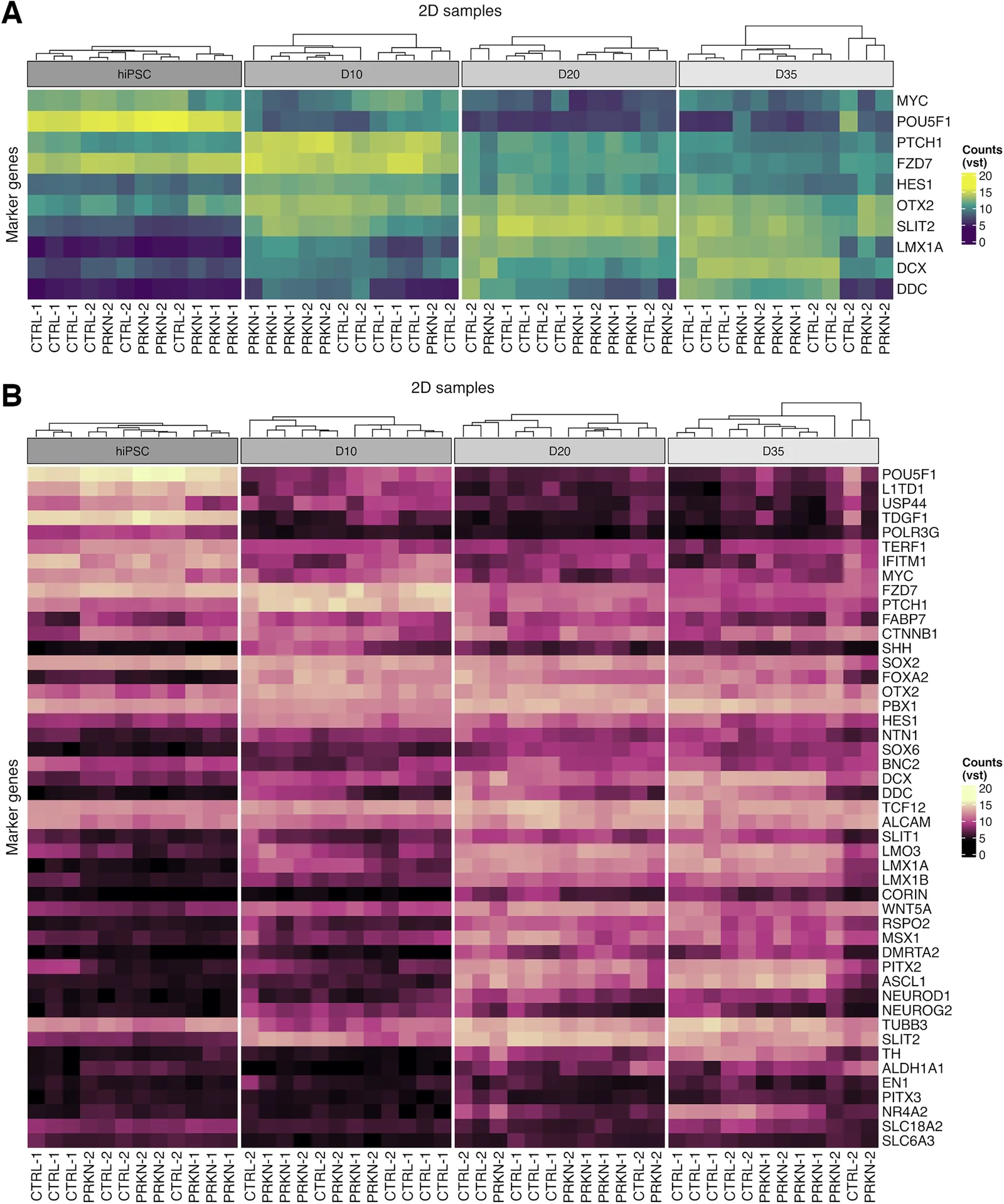
Validation of mDA identity in hiPSC-derived neurons from healthy controls and *PRKN* mutation carriers generated using the 2D culture method. **A** Heatmap showing the transition in gene expression during differentiation from hiPSCs to mDA neurons across D10, D20, and D35 in all four samples of the 2D dataset. Early pluripotency markers (*MYC*, *POU5F1 – OCT4*) progressively decrease, whereas genes associated with mDA specification and differentiation (*PTCH1*, *FZD2*, *HES1*, *OTX2*, *SLIT2*, and *LMX1A*) and early mature neuronal markers (*DCX*, *DDC*) increase over time. Each column represents a specific differentiation timepoint for control or *PRKN*-mutant cells, and each row corresponds to the expression of an individual gene. Colors indicate variance-stabilized count data, essentially log_2_-transformed raw counts. **B** Heatmap showing a more extensive set of marker genes reported to be relevant and necessary for mDA differentiation evaluated in the 2D dataset for all four samples. These markers were selected to recapitulate the in vivo developmental trajectory of mDA neurons, spanning multiple transitional stages, including hiPSCs, radial glia, neural progenitors, and lineage-committed precursor populations of the developing midbrain. Because several markers are expressed across overlapping developmental stages and cell identities, genes were ordered empirically to reflect progression along the mDA differentiation trajectory rather than grouped into discrete cell type categories. Both panels were adapted from differentiation stage representations described by Novak et al.^29^. *n* = 3 independent differentiations.

To verify that both the 2D and 3D differentiation protocols used are able to recapitulate the in vivo mDA developmental process and that the derived neurons displayed a characteristic mDA phenotype, we evaluated the expression of genes known to be involved and necessary for mDA neuron differentiation^19,22–24^, which differs from other DA neurons. During early neural development, mDA neurons arise from radial glial cells of the floor plate, and their development is strictly determined by exposure to high levels of SHH transcription factor^25^, followed by the induction of Wnt signaling and the expression of a specific mDA downstream identity^19,23,26,27^. For both differentiation approaches and all four cell lines, a clear transition in gene expression from hiPSC markers (*MYC* and *POU5F1-OCT3/4*) to genes associated with mDA neuron differentiation (*PTCH1*, *FZDZ*, *HES1*, *OTX2*, *SLIT1*, and *LMX1A*) and finally to an early expression of mature mDA markers (*DCX* and *DDC*) was observed (Fig. 3A and Supplementary Fig. 3A). To provide a more comprehensive analysis of the neuronal differentiation process, we generated a detailed gene list of key regulators expressed at different stages of human mDA differentiation^28,29^. Consistent with in vivo differentiation steps, both in 2D and in 3D, *PTCH1*, a receptor for SHH, and *FZD7*, a receptor for Wnt proteins, were the most highly expressed genes at D10 of differentiation. At D20 of differentiation, we observed high expression levels of specific mDA differentiation genes, such as *TCF12*, *ALCAM*, *OTX2*, *LMX1A*, *DCX*, and *WNT5A*. At D35 of differentiation, high expression levels were observed for *PBX1*, which is required for the morphological maturation of DA neurons, and *LMO3*, a transcriptional co-activator of *PITX3* (Fig. 3B and Supplementary Fig. 3B). Moreover, a gene set enrichment analysis of DEGs between hiPSC and D35 in both control- and patient-derived cells was performed using the gene set signatures for different cell types identified in single-cell sequencing studies of human tissue from The Molecular Signatures Database (MSigDB)^30^. At D35, hiPSC-derived neurons from both controls and *PRKN* mutants displayed a clear enrichment for midbrain-related gene sets in both the 2D and 3D culture conditions (Supplementary Fig. 4A, B).

### Identification of a molecular signature of *PRKN*-PD using the 2D neuronal cultures

**Fig. 4:**
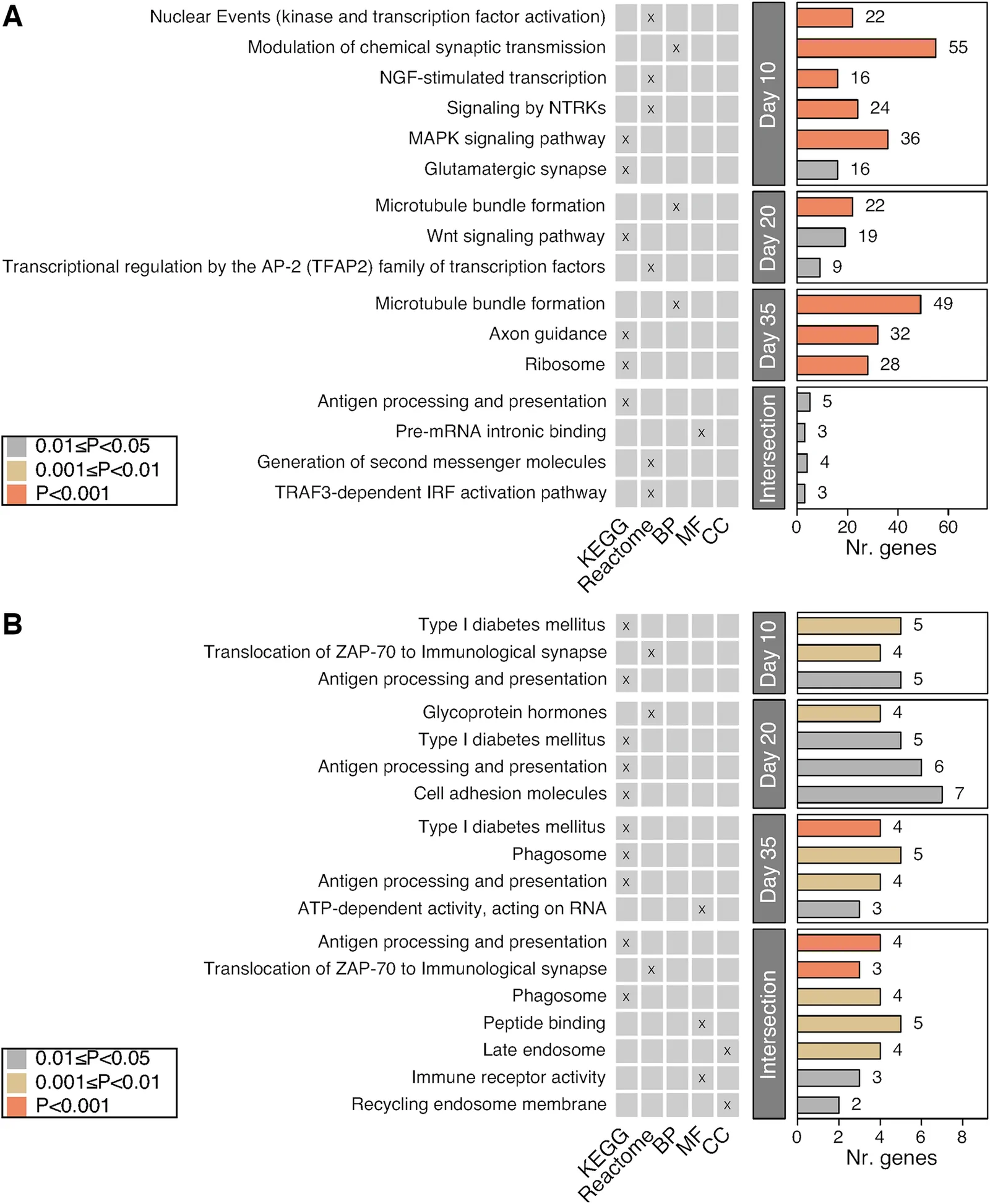
Functional annotation analysis of the DEGs altered across all three timepoints and at each single timepoint in the 2D and 3D culture conditions. **A** Names of selected, over-represented pathways or over-represented GO terms at the single timepoints (D10, D20, and D35 of differentiation) and across all three timepoints in the 2D culture condition are shown on the left side. A checked box indicates the origin of the pathways (KEGG, Reactome) or GO ontologies (biological process, BP; molecular function, MF; cellular component, CC). The horizontal bars on the right side represent the number of overlapping, significantly dysregulated genes (numbers shown next to bar) and are colored by *p* value obtained from the pathway or GO term over-representation analysis (see legend for coloring scheme). **B** Like **A**, but for the 3D culture condition. *n* = 3 independent differentiations.

To gain further insight into the molecular mechanisms affected by the DEGs between the *PRKN* mutation carriers and controls, enrichment analysis of the 2D DEGs altered in the same direction at all three timepoints (*n* = 151 genes) was performed. Interestingly, functional annotation analysis using GO, KEGG, and Reactome revealed high enrichment of biological terms related to antigen processing and presentation (hsa04612), generation of second messenger molecules (R-HSA-202433), TRAF3 (TNF receptor-associated factor 3)-dependent IRF (interferon regulatory factors)-transcription factor activation pathway (R-HSA-918233), and pre-mRNA intronic binding (GO:0097157) (Fig. 4A). *RFX5* (regulatory factor X5) (hsa04612), *PAK1* (p21-activated serine/threonine kinase 1) (R-HSA-202433), *CREBBP* (CREB-binding protein) and *EP300* (E1A-binding protein p300) (R-HSA-918233), and *ELAVL4* (HuD) (GO:0097157) were among the genes that contributed to the enriched pathways.

Next, the functional annotations associated with the DEGs at the single timepoints (D10, *n* = 937; D20, *n* = 819; D35, *n* = 1425) were assessed. D10 yielded high enrichment of genes contained in the MAPK signaling pathway (hsa04010), glutamatergic synapse (hsa04724), nuclear events-kinase and transcription factor activation (R-HSA-198725), nerve growth factor (NGF)-stimulated transcription (R-HSA-9031628), signaling by NTRKs (neurotrophic tyrosine receptor kinase) (R-HSA-166520), and modulation of chemical synaptic transmission (GO:0050804) (Fig. 4A). At D20 of differentiation, we identified the Wnt signaling pathway (hsa04310), transcriptional regulation by the AP-2 (activating enhancer-binding protein 2) family of transcription factors (R-HSA-8864260), and microtubule bundle formation (GO:0001578) among the key hits from GO, KEGG, and Reactome (Fig. 4A). At D35 of differentiation, we observed a prioritization of axon guidance (hsa04360), ribosome (hsa03010), and microtubule bundle formation (GO:0001578) annotated pathways (Fig. 4A).

### Identification of key regulatory mechanisms of *PRKN*-PD using the hydrogel-based 3D system

Next, we analyzed the transcriptional profile of the *PRKN* mutant lines and controls obtained by using the Alg/Fn-based 3D protocol^20^. Enrichment analysis of DEGs across all three timepoints using KEGG, Reactome, and GO database resources highlighted a correlation between the *PRKN* gene mutations and alteration of immune response pathways, as well as pathways related to phagosome and late endosome function (Fig. 4B). In addition to a significant upregulation of *CD200R1* (CD200 receptor 1), the microglial inhibitory receptor, expressed at very low levels in our cultures (Supplementary Fig. 5), a downregulated expression of *WASHC1* (WASH complex subunit 1) and *RAPGEF2* (Rap guanine nucleotide exchange factor 2), which regulate endosomal trafficking and synaptic remodeling and neural plasticity, were detected. By analyzing the single timepoints, an enrichment of functional annotations linked to type I diabetes mellitus (hsa04940) and immune system (hsa04612, R-HSA-202430) was observed at D10 of differentiation (Fig. 4B). Notably, *ZAP70* (R-HSA-202430), which encodes zeta-chain-associated protein kinase 70 that is essential for the regulation of the adaptive immune response, and *PTPRN2* (protein tyrosine phosphatase receptor type N2) (hsa04940), which plays a role in insulin secretion in response to glucose stimuli and is required for normal accumulation of the neurotransmitters norepinephrine, dopamine, and serotonin in the brain, were listed among the genes contributing to the enrichment in these pathways. At D20, type I diabetes mellitus (hsa04940), antigen processing and presentation (hsa04612), cell adhesion molecules (hsa04514), and glycoprotein hormones (R-HSA-209822) were dysregulated in the *PRKN* mutation carriers (Fig. 4B). Finally, at D35, alterations in the following pathways were detected: type I diabetes mellitus (hsa04940), phagosome (hsa04145), antigen processing and presentation (hsa04612), and ATP-dependent activity, acting on RNA (GO:0008186) (Fig. 4B). Among the altered genes, dysregulation of *ATP6V0A4* (hsa04145), which encodes a subunit of vacuolar ATPase, and *DDX3Y* (GO:0008186), which encodes a cytoplasmic RNA helicase (DEAD-box helicase 3, Y-linked) that promotes translation of mRNAs with highly structured 5′ UTRs, were observed. Notably, the expression of several HLA-related genes was downregulated in the *PRKN* mutation carriers compared to the controls, across the entire dataset and at the single timepoints of the differentiation process.

### Patient-specific transcriptomic profile in the integrated 2D and 3D culture systems

**Fig. 5:**
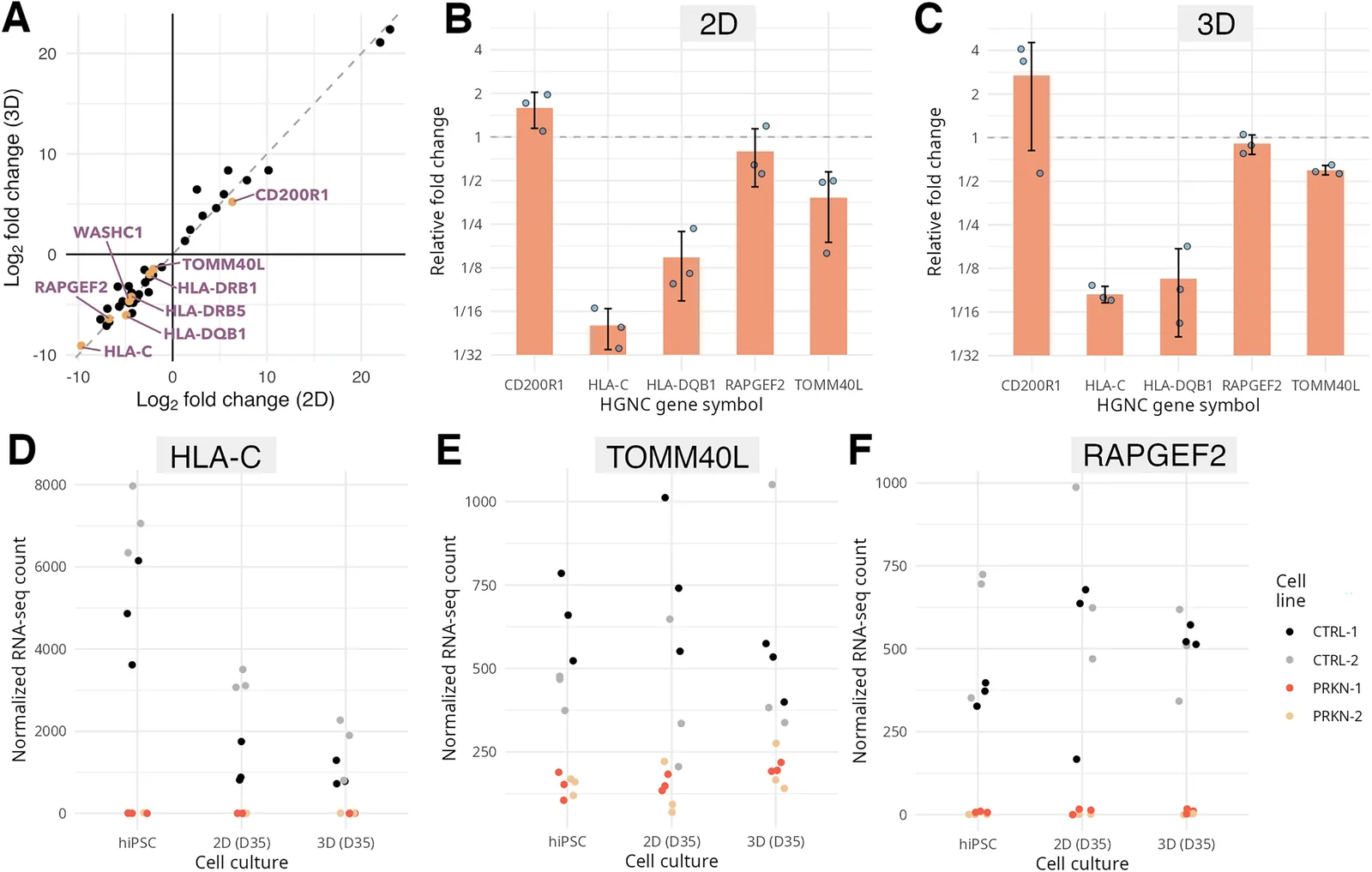
Comparison of fold change of 2D and 3D culture systems at D35. **A** Shown here is a scatter plot of the log_2_ fold change of significantly dysregulated genes detected in the 2D culture system (*x*-axis) and in the Alg/Fn culture system (*y*-axis) at D35. The diagonal, indicating equal fold change for both culture systems, is shown as a gray dashed line. Log_2_ fold changes of genes highlighted in orange are discussed in the main text and are plotted separately in (**D**–**F**) and Supplementary Fig. 5. **B**, **C** qRT-PCR analysis of *CD200R1*, *HLA-C*, *HLA-DQB1*, *RAPGEF2*, and *TOMM40L* in the *PRKN*-1 line compared to CTRL-1 at D35 of differentiation in both 2D and 3D conditions. Points are single measurements representing 2^-ΔΔCT^, the height of the bars is equal to the mean of each of the three measurements, and the error bars extend by 1 unit of standard error in both directions. The *y*-axis is on a log_2_ scale. **D**–**F** Plot of RNA-seq count data normalized by estimated size factor (DESeq2 function plotCounts) for *HLA-C*, *TOMM40L*, and *RAPGEF2* for both *PRKN*-mutant and CTRL lines in 2D and 3D conditions at hiPSC and day 35. *n* = 3 independent differentiations.

To further elucidate pathway alterations in *PRKN* mutants, we analyzed the DEGs across both the 2D and 3D datasets. This combined gene expression signature, consistent across culture methods, is likely to be more relevant to recapitulate the disease process. In this integrated dataset, expression of 40 genes was altered in *PRKN* mDA neurons at D35 of differentiation (Supplementary Data 2). Overall, the enriched biological processes associated with these dysregulated genes align with pathways implicated in PD pathogenesis, including immune regulation, mitochondrial function, and endo-lysosomal trafficking^31–33^ (Supplementary Data 2). Among the DEGs contributing to immune-related pathways, several *HLA* genes, the microglial inhibitory receptor *CD200R1*, and *TOMM40L* (translocase of outer mitochondrial membrane 40 like) were identified (Fig. 5A). Expression of the major histocompatibility (MHC) class I gene *HLA-C* was reduced in *PRKN* mutation carriers, accompanied by decreased expression of MHC class II genes *HLA-DQB1, HLA-DRB1*, and *HLA-DRB5*, while low-level *CD200R1* expression was increased. *TOMM40L*, a paralog of *TOMM40* encoding a component of the mitochondrial import machinery, was also significantly downregulated in *PRKN* mutation carriers, further supporting the involvement of mitochondrial dysfunction in *PRKN*-PD (Fig. 5 and Supplementary Fig. 5). In addition, dysregulation of the autophagy-lysosomal pathway, central to both idiopathic and familial PD, was reflected by reduced expression of *WASHC1* and *RAPGEF2*, both associated with endo-lysosomal compartments (GO:0005770: late endosome and GO:0030139: endocytic vesicle) (Fig. 5 and Supplementary Figure 5). *WASHC1* regulates endosomal trafficking and autophagosome formation^34,35^, and *RAPGEF2* plays a key role in synaptic remodeling and neural plasticity^36^.

Five major DEGs (*HLA-C*, *HLA-DQB1*, *TOMM40L*, *CD200R1*, and *RAPGEF2*) were chosen for validation by qRT-PCR analysis to confirm the RNA-seq findings in neuronal differentiations of *PRKN*-1 and CTRL-1 cell lines. For all genes, the direction of the effect was confirmed in both 2D and 3D conditions (Fig. 5B, C).

In addition, to investigate whether inflammatory activation influences *HLA-C* expression in a human neuron-microglia co-culture system in the context of *PRKN* mutations, we analyzed RNA samples extracted from hiPSC-derived co-cultures from controls (*n* = 4) and patients with *PRKN* mutations (*n* = 3), exposed to LPS, mtDNA, or both, previously generated and characterized by Wasner et al.^15^. Responsiveness to inflammatory stimuli was confirmed by increased IL6 expression following combined LPS and mtDNA treatment, with a stronger response observed in *PRKN*-mutant co-cultures^15^. Expression of the pan-neuronal marker *MAP2* and the microglial marker *AIF1* (*IBA1*) in these co-cultures is shown in Supplementary Fig. 6A. Using these same RNA samples, we analyzed *HLA-C* expression and found that inflammatory stimulation did not induce *HLA-C* expression in either genotype. Moreover, no significant difference in *HLA-C* expression was observed between *PRKN*-mutant and control cultures (Supplementary Fig. 6B).

### Overlapping DEGs of 2D and 3D culture with MitoCarta and GWAS data

To further explore the biological significance of the identified transcriptional modifications in mDA neurons with *PRKN*-mutations and their direct or indirect relation to mitochondrial processes, the DEGs of the 2D and 3D culture datasets at D35 of differentiation were intersected with the 1136 mitochondrial genes listed in the Human MitoCarta 3.0 project (human genes selected for high confidence of mitochondrial localization based on integrated proteomics, computation, and microscopy, including manually curated annotations of sub-mitochondrial localization (matrix, inner membrane, intermembrane space, and outer membrane) and assignment to 149 hierarchical “MitoPathways” spanning seven broad functional categories relevant to mitochondria^37^). For the 2D dataset, 43 DEGs overlapped with the MitoCarta project dataset (Supplementary Data 3), which clustered mainly in three mitochondrial pathways: metabolism, central dogma, and small molecules transport. The three most downregulated genes in the *PRKN* mutation carriers were *TOMM40L, STARD7* (StAR-related lipid transfer domain containing 7), and *GLUD2* (glutamate dehydrogenase 2). Interestingly, *CHCHD2*, which encodes the coiled-coil-helix-coiled-coil-helix domain-containing protein 2, was among the upregulated genes. Mutations in *CHCHD2* have been linked to PD and are associated with mitochondrial network fragmentation and impaired oxidative phosphorylation at complexes I and IV^38,39^.

In the 3D dataset, we identified an overlap with the Human MitoCarta 3.0 data for three downregulated genes (*TOMM40L, GLUD2*, and *SDHC*, encoding succinate dehydrogenase complex subunit C), involved in mitochondrial metabolism, oxidative phosphorylation, and small molecules transport. Downregulation of *SDHC* was already reported in an integrated meta-analysis of multiple gene expression array datasets as a candidate target gene in PD^40^. No upregulated genes were identified (Supplementary Data 3).

Furthermore, 21 DEGs in the *PRKN* mutants in the 2D cultures overlapped with significant results from the largest genome-wide association study (GWAS) for PD^41^. The top three differentially expressed overlapping genes were *CHL1* (cell adhesion molecule L1-like), *STX1B* (Syntaxin 1B), and *IGSF9B* (immunoglobulin superfamily member 9B). For the 3D culture condition, an overlap of *HLA-DQB1* and *HLA-DRB1* was identified.

## Discussion

PD is broadly accepted as a multifactorial disorder, with both genetic and environmental factors playing an important role, and age being the biggest risk factor for developing the disease^1,42^. Important pathophysiological clues have been provided from the monogenic forms of PD, making the identification of PD signatures driven by familial PD genes particularly vital to portray the molecular heterogeneity of PD.

Mutations in the *PRKN* gene represent the most common known cause of autosomal recessive early-onset PD and lead to misfolded and mislocalized Parkin, resulting in a loss of function as ubiquitin ligase. Overall, Parkin has been characterized as a multifunctional protein involved in many cellular processes, including control of mitochondrial integrity, mitophagy, regulation of apoptosis, transcription, and synaptic function^21,43^. Investigating the transcriptional profile in patient neurons is crucial to reveal molecular alterations underlying PD pathophysiology, especially because *post-mortem* brain samples predominantly represent end-stage disease and therefore provide limited insight into the early processes that precede neuronal loss^44^. Human iPSC-derived neuronal models can bridge this gap by providing early phenotypes of PD pathology^29^. The RNA-seq analysis performed in this study took advantage of combining two differentiation approaches for the generation of mDA neurons from hiPSCs, a 2D method^18^ and a hydrogel-based 3D model system recently developed by our laboratory^20^. Both 2D and 3D hiPSC-derived in vitro culture systems offer two major advantages: first, they enable the investigation of early disease-relevant processes, and second, they avoid species-specific differences inherent to animal models, providing a genetically human context for mechanistic studies. A crucial aspect for the application of an in vitro model system for disease modeling is its ability to recapitulate the in vivo phenotype and processes. We have therefore verified that both differentiation methods were able to mimic the in vivo mDA differentiation path. Since the expression of tyrosine hydroxylase (*TH*) alone is not sufficient to prove the dopaminergic nature of neurons^24^, the expression of key genes associated with specific stages of the neuronal differentiation, including stemness, mDA differentiation, and mature mDA neuron markers, was used to define the transition profile during differentiation from hiPSCs to radial glia, neuroprogenitors, and finally early mature mDA neurons^45^. In both models, the analysis of the four timepoints (hiPSC, D10, D20, and D35) revealed the expression of these specific differentiation stages, confirming their validity.

In a previously performed detailed comparison of the 2D and 3D culture methods, we found that DA neurons generated with both methods reached a relatively advanced functional level of in vitro maturity by day 35, and the 3D neurons reached this level of maturity earlier during differentiation^20^.

To analyze the DEGs in our transcriptomics dataset, we used multiple approaches that revealed a large number of gene expression alterations in neurons derived from *PRKN* patients compared to controls. By restricting our analysis to genes that were significantly altered across all three timepoints (D10, D20, and D35), the contribution of genes whose expression changes during mDA differentiation was minimized. In this dataset of 2D culture condition, we found *RFX5* (hsa04612), a transcription factor influencing MHC class II expression^46^, downregulated at all three timepoints. Additional downregulated genes included *PAK1* (R-HSA-202433), *CREBBP* and *EP300* (R-HSA-918233). Oxidative stress-induced downregulation of *PAK1* activity has previously been associated with neurodegenerative diseases and may contribute to the loss of mesencephalic dopaminergic neurons^47^. *CREBBP* and *EP300* encode closely related lysine acetyltransferases that regulate transcription and DNA repair by binding to acetylated lysine residues. Dysregulated acetylation has been implicated in protein misfolding and aggregation^48^, and inhibition of CBP/p300 bromodomain has been reported to decrease amyloid-like aggregates and cytotoxicity^48,49^.

Enrichment analysis of the *PRKN* transcriptomics signature in the hydrogel-based 3D neuronal cultures across all three timepoints revealed significant associations with immune response pathways, in particular antigen processing and presentation. The consistent enrichment of the phagosome pathway in the 3D cultures further confirmed a role of endosomal sorting pathways in *PRKN*-PD pathogenesis. Functional annotation analyses performed separately at each timepoint reinforced this pattern, identifying dysregulation of immune-related pathways, type I diabetes mellitus, glycoprotein hormones, cell adhesion molecules and the phagosome pathway. While inflammatory responses are observed in many diseases and are often regarded as a secondary effect, recent research suggests that neuroinflammatory processes may act as causative or contributing factors in PD^50^ and *PRKN*-PD^51^. Interestingly, epidemiological studies indicate that individuals with diabetes have an increased risk of developing PD^52^. Beyond the central nervous system, Parkin contributes to mitochondrial homeostasis in peripheral tissues, such as the liver and has been shown to exert a protective role against insulin resistance^53^, suggesting a potential link between metabolic regulation and *PRKN*-associated disease mechanisms. Moreover, recent studies have reported PD-associated, sex-specific DNA methylation changes in *PTPRN2 (*protein tyrosine phosphatase, receptor type N2)^54^, a gene involved in glucose-stimulated insulin secretion and genetically linked to type I diabetes^55^.

Together, these findings raise the possibility that metabolic and immune-regulatory pathways intersect in *PRKN*-PD, extending beyond classical mitochondrial dysfunction. Further studies are required to clarify this potential relationship and to determine whether modulation of metabolic pathways—including the possible repurposing of antidiabetic therapies—may hold therapeutic relevance in *PRKN*-PD.

Notably, expression of several *HLA* genes, including *HLA-C*, *HLA-DRB1, HLA-DRB5*, and *HLA-DQB1,* was consistently reduced in *PRKN* mutation carriers compared to controls. Given that our cultures are largely neuronal, lack detectable microglial markers, and show only minimal expression of oligodendrocyte and astrocyte lineage markers, it is highly unlikely that the observed HLA expression changes originate from immune cells, such as microglia. Reduced expression of *HLA-C*, an MHC class I molecule known to be expressed by neurons^56^, is therefore consistent with altered neuron-intrinsic regulation. In contrast, the low-level expression and downregulation of MHC class II genes (*HLA-DQB1*, *HLA-DRB1*, and *HLA-DRB5*), which are not typically expressed by neurons under baseline conditions, likely reflect contributions from minor non-immune glial populations, most plausibly astrocytes. Together, these findings indicate that *PRKN* deficiency alters HLA gene expression in neuron-enriched cultures.

Notably, although loss of Parkin has been shown to enhance mitochondrial antigen presentation via MHC class I in immune cells^16^, it remains unclear whether this mechanism operates similarly in non-immune cells, like neurons. HLA gene expression and antigen presentation capacity may be differentially regulated in neurons, indicating that Parkin-dependent effects on immune signaling are likely to be cell type-specific. To further address this question, we have analyzed *HLA-C* expression in hiPSC-derived neuron-microglia co-cultures from *PRKN* patients and controls following exposure to LPS, mtDNA isolated from patients with *PRKN*-PD or both. These inflammatory stimuli did not induce *HLA-C* expression in either genotype, and no differences were observed between *PRKN*-mutant and control cultures, arguing against a classical immune activation mechanism. We therefore considered whether reduced HLA-C expression might instead reflect altered neuron-intrinsic functions of MHC class I proteins.

Beyond their established immune function, MHC class I proteins are known regulators of axon regeneration and activity-dependent synaptic pruning^57^. Notably, synaptic pruning depends on the intracellular domain of MHC-I, suggesting that downstream intracellular signaling contributes to this process^58^. Thus, reduced *HLA-C* in *PRKN* neurons may indicate impaired synaptic plasticity or maturation. In line with this interpretation, Parkin has previously been implicated in early synaptic dysfunction^59^. Supporting this hypothesis, several additional DEGs identified in our dataset are linked to synaptic maintenance and remodeling. *WASHC1* and *RAPGEF2*, both associated with lysosomal and endosomal compartments (GO:0005770; GO:0055038), were consistently downregulated. *WASHC1*, a core component of the WASH complex, coordinates endosomal trafficking, which is essential for maintaining synaptic function, structure, and plasticity, and autophagosome formation^34,35^. *RAPGEF2* plays critical roles in synaptic remodeling and neural plasticity and has been implicated in Aβ oligomer-induced synaptic and cognitive impairments in the AD hippocampus^36^. Together, these alterations point toward disrupted vesicular trafficking and synaptic homeostasis in *PRKN* neurons.

In addition, we also observed increased expression of *CD200R1*, which has been reported to be elevated in the hippocampus of PD patients^60^, suggesting altered CD200-CD200R1 signaling and potential modulation of microglial activity.

When comparing the DEGs at D35 to the genes listed in the Human MitoCarta 3.0 database, *TOMM40L*, a paralog of *TOMM40*, was the most significantly downregulated gene in the *PRKN* mutation carriers. Paralogous proteins often retain similar or overlapping functions. The translocase of the outer mitochondrial membrane (TOMM) is a multiprotein complex essential for importing most mitochondrial proteins, which consists of seven different subunits: the receptors Tom20, Tom22, and Tom70, the channel protein Tom40, and three small Tom proteins involved in the assembly and dynamics of the TOM complex, Tom5, Tom6, and Tom7^61^. PD-causing *PRKN* mutations weakened or disrupted the interaction between Parkin and the surface receptor TOMM70A and the channel protein TOMM40^62^. Interestingly, mutant *TOMM40* has previously been shown to induce mitochondrial dysfunction and oxidative stress-mediated microglial activation in AD^63,64^. *GLUD2*, which encodes a mitochondrial protein crucial for glutamate recycling during neurotransmission, was also downregulated. The most strongly upregulated gene was *CHCHD2*, which, together with *CHCHD10*, localizes to the intermembrane space of the mitochondria and regulates mitochondrial homeostasis. CHCHD2 has been reported to also function as a nuclear transcription factor regulating a cytochrome c oxidase subunit and CHCHD2 itself, thereby influencing mitochondrial respiration, mitochondrial cristae structure, and apoptosis in response to hypoxic stress^65^. Mutations in the *CHCHD2* gene cause an autosomal dominant form of late-onset PD that closely resembles iPD^38^. Recent work using a *CHCHD2* T61I knock-in mouse model has demonstrated pronounced mitochondrial abnormalities in *substantia nigra* DA neurons, including disrupted cristae structure, CHCHD2 aggregation, elevated mitochondrial ROS, impaired respiratory function, and progressive α-synuclein accumulation. Moreover, in iPD, CHCHD2 expression correlates with α-synuclein levels in DA neurons. These findings suggest that CHCHD2 accumulation may contribute to mitochondrial dysfunction and promote α-synuclein aggregation^66^. Given the central role of Parkin in mitochondrial function, increased CHCHD2 expression may represent a convergent node linking mitochondrial stress, oxidative damage, and possibly α-synuclein-associated pathology in *PRKN*-associated PD.

While both 2D and 3D neuronal cultures showed overlapping alterations in these identified biological processes, certain genes and pathways were uniquely affected in only one of the two models. For instance, alterations related to type I diabetes mellitus were detected exclusively in the 3D cultures. This observation aligns with our previous characterization of the 3D Alg/Fn-neuronal system, which suggested that it represents a more complex and mature neuronal environment compared to conventional 2D cultures^20^. This increased complexity and maturity may underly the model’s ability to reveal additional or earlier phenotypic changes. Indeed, we found that some pathway-level transcriptomic alterations emerged earlier during differentiation in the 3D cultures than in the 2D system, highlighting the temporal and biological sensitivity of the 3D model.

Overall, the extensive dysregulation of genes in *PRKN* mutation carriers underscores critical pathways affected in *PRKN*-linked PD, many of which are also implicated in iPD. The results suggest that *PRKN* mutations act through interconnected rather than through separate, parallel mechanisms. Notably, many of the genes and pathways are consistently dysregulated at all investigated timepoints during neuronal differentiation, including the earlier timepoints (D10 and D20), likely reflecting early events in the disease development. Therefore, these alterations could serve for the development of diagnostic markers and targets for prevention and early intervention in the disease process.

## Methods

### Cell lines

Human iPSCs from two control individuals (CTRL-1: hiPS-SFC084-03-02 and CTRL-2: hiPS-802) and two PD patients carrying a biallelic mutation in the *PRKN* gene (*PRKN*-1: hiPS-B125 and *PRKN*-2: hiPS-2011) were used. *PRKN*-1 carries a homozygous 1-bp deletion in exon 9 (p.Val324AlafsTer111, rs1562519380), and *PRKN*-2 carries a homozygous deletion of exon 3. hiPSC-802 and hiPSC-2011^67^ lines were generated in the laboratory of the Institute for Biomedicine according to a previously published protocol^68^. The control hiPSC line SFC084-03-02 was established through the StemBANCC consortium (https://cells.ebisc.org/STBCi033-B/), and hiPSC-B125 was previously generated at the Institute of Neurogenetics of the University of Lübeck^21^. Phenotypic and genotypic information for the hiPSC lines are described in Supplementary Table 1. The study was approved by the ethics committee of the South Tyrolean health care system (approval number 102/2014). The participants provided their written informed consent, and all ethical regulations relevant to human research participants were followed.

### Human iPSC culture

hiPSCs were cultured under feeder-free conditions on Matrigel matrix (Corning) in StemMACS iPS-Brew XF (StemMACS) with 1% penicillin-streptomycin (Gibco). Cells were maintained in a saturated humidified atmosphere at 37 °C and 5% CO_2_, and medium was changed every day. hiPSC colonies were passaged enzymatically using 1 mg/ml collagenase type IV (Gibco) in DMEM/F12 (Gibco).

### Generation of 3D cultures

Encapsulation of hiPSCs in alginate/fibronectin (Alg/Fn) beads was performed as we have described recently^20^. In brief, hiPSCs were gently mixed with the Alg/Fn solution, and beads were formed by slowly dripping the prepared Alg/Fn cell suspension in a 0.1 M CaCl_*2*_ solution with a 19-gauge needle. Beads were then incubated for 20 min in the CaCl_2_ solution and washed three times with 1× PBS pH 7.4. Subsequently, cell-loaded beads were transferred to 12-well cell culture plates (10–12 beads/well) and supplied with StemMACS iPS-Brew XF medium.

### Dopaminergic neuronal differentiation in 2D and 3D conditions

For 2D monolayer differentiation, 3 × 10^4^ cells/cm^2^ were seeded on 12-well cell culture plates coated with Matrigel (Corning) in StemMACS iPS-Brew XF medium supplemented with 10 μM ROCK inhibitor Y-27632, and the differentiation of hiPSCs into mDA neurons was initiated once the cells reached 90% confluence. The differentiation both in 2D and 3D conditions was initiated following the floor-plate-based neuron induction protocol established by Kriks et al., with minor modifications^18,20,21,69^. In brief, differentiation was started by supplying the cells with knockout serum replacement (KSR) medium, knockout DMEM, 15% knockout serum, L-glutamine (2 mM), nonessential amino acids (1×), β-mercaptoethanol (100 µM), penicillin/streptomycin (1×) (all Gibco), supplemented with SMAD pathway inhibitors SB431542 (SB, StemMACS, 10 µM) and LDN-193189 (LDN, StemMACS, 100 nM). LDN was added on days 1–10, while the supplementation with SB was stopped on day 6. On days 2–7, recombinant Human Sonic Hedgehog (SHH, R&D System, 100 ng/ml), recombinant Human FGF-8a (FGF8, R&D System, 200 ng/ml), and purmorphamine (Pu, StemMACS, 2 µM) were added to the medium. Furthermore, on days 4–12, the Wnt pathway activator molecule CHIR99021 (CH, StemMACS, 3 µM) was added to the differentiation medium. During days 7–10 of differentiation, increasing amounts of neurobasal medium (Gibco) plus B27 supplement (Gibco) were added to the KSR medium (25, 50, and 75%). On day 12, maturation of mDA neurons was initiated by adding recombinant Human BDNF (Peprotech, 20 ng/ml), recombinant Human GDNF (Peprotech, 20 ng/ml), ascorbic acid (AA, Sigma-Aldrich, 0.2 mM), recombinant Human TGF-beta 3 (ß3, Peprotech, 1 ng/ml), dibutyryl-cyclic-AMP (dbcAMP, Sigma-Aldrich, 0.5 mM), and DAPT (Tocris, 10 µM). On day 20 of differentiation, cells differentiated in 2D were passaged *en bloc* or as single cells on 24-well plates coated with poly-D-lysine (Sigma-Aldrich) and Laminin (Sigma-Aldrich) (PDL/LA).

### Generation and treatment of hiPSC-derived neuron-microglia co-cultures

Midbrain dopaminergic neurons and microglia cells were generated according to established protocols^18,70^. On day 92 of neuronal differentiation, microglia precursor cells were plated onto corresponding cell lines of neurons. Equal amounts of both cell types were co-cultured for 35 days before treatment with inflammatory stimuli. Co-cultures were derived from controls (*n* = 4) and patients with *PRKN* mutations (*n* = 3), exposed to LPS (100 ng/ml) for 3 h and mtDNA (300 ng) for 1 h as described by Wasner et al.^15^. We have used RNA-samples extracted from these co-cultures described and analyzed by Wasner et al.^15^ for our study. Gene expression levels of *MAP2* and *AIF1 (IBA1*) were determined quantitatively on the Lightcycler 480 (Roche) using Maxima SYBR Green/Fluorescein qPCR Master Mix (Thermo Fisher Scientific) using the following primers: *MAP2* forward 5′ CAGGTGGCGGACGTGTGAAAATTGAGAGTG-3′ and reverse 5′-CACGCTGGATCTGCCTGGGGACTGTG-3′; *AIF1* (*IBA1*) forward 5′-GACCTTAATGGAAATGGCGATA-3′ and reverse 5′-ATCTCTTGCCCAGCATCATC-3′.

### RNA extraction

Samples for RNA isolation were collected on days 0 (hiPSC), D10, D20, and D35 of neuronal differentiation. In the 3D cultures, cells were recovered from the beads by washing with PBS without Ca^2+^ and Mg^2+^ and centrifugation at the same timepoints as for 2D cultures. Total RNA was isolated using the Direct-zol RNA Miniprep Kit (#R2052, Zymo research), following manufacturer’s instructions. RNA integrity was assessed on an Experion Electrophoresis Station (Bio-Rad).

### Quantitative reverse-transcription PCR (qRT-PCR)

RNA was reverse transcribed using the SuperScript® VILO™ cDNA Synthesis Kit (Thermo Fisher Scientific) following the manufacturers’ protocol. qRT-PCR was performed in triplicate on a CFX96 Real-Time PCR Detection System (Bio-Rad) in 10 µl final volume using the TaqMan™ Gene Expression Master Mix (Thermo Fisher Scientific) and the following TaqMan gene expression assays: hs05049205_g1 (*HLA-C*), Hs03054971_m1 (*HLA-DQB1*), Hs00375646_g1 (*TOMM40L*), Hs00793597_m1 (*CD200R1*), and Hs01127851_m1 (*RAPGEF2*) from Thermo Fisher Scientific. The gene expression levels were normalized to the housekeeping gene *RPP30* (Human HEX IDqHsaCEP0052683, Bio-Rad) using the ΔΔCt method.

### Western blot analysis

For Western blot analysis, whole cell homogenates were used. Harvested cells were resuspended in cold RIPA buffer (Thermo Fisher Scientific) supplemented with cOmplete™ Protease Inhibitor Cocktail (Roche) and PhosSTOP™ phosphatase inhibitors (Roche). Protein concentrations were assessed using BCA Protein Assay Kit (Thermo Fisher Scientific), and 10 µg of total protein lysates were loaded per well on a NuPAGE 4–12% Bis-Tris SDS-PAGE gel (Thermo Fisher Scientific). After electrophoresis, proteins were transferred onto a PVDF membrane (Bio-Rad) and probed with antibodies against Parkin (Park8, Abcam Cat# ab77924, RRID:AB_1566559, 1:1000) and GAPDH (Millipore Cat# MAB374, RRID:AB_2107445, 1:1000). Subsequently, the blots were incubated with the corresponding secondary antibodies, and target proteins were detected by enhanced chemiluminescence using Clarity™ ECL Western Kit (Bio-Rad). The chemiluminescence signal was detected using the ChemiDoc™ Touch Imaging System (Bio-Rad). The original, unedited Western blot images are shown in Supplementary Fig. 7.

### Statistics and reproducibility

Targeted RNA-seq libraries were produced using the DriverMapT Human Genome-Wide Expression Profiling Kit, V2 (Cellecta), which covers all 19,000 human protein-coding genes. All experiments were performed in three independent biological replicates (three neuronal differentiations), except for the analyses presented in Supplementary Fig. 6, for which one differentiation was performed. All samples were sequenced in 38 bp paired-end reads on the Illumina NextSeq500 platform by Personal Genomics, Verona (Italy). Raw reads (fastq) were checked using FastQC (v0.11.9) (www.bioinformatics.babraham.ac.uk/projects/fastqc/) and MultiQC (v1.09)^71^. Transcripts were aligned with STAR (v 2.7.10a)^72^ to the human transcriptome GRCh38 (Ensembl v100) and quantified with featureCounts (package Rsubread v2.10.4)^73^. All statistical analyses were performed in the R statistical programming language (v4.2.0, Bioconductor v3.15). Differential expression analysis was performed with the DESeq2 package (v1.28.1)^74^ using the Wald test for significance testing. Principal component analysis (PCA) was run with the plotPCA function included in the DESeq2 package. For heatmap plotting, raw counts were transformed with the vst variance stabilizing transformation function from DESeq2 with default parameters, which in essence performs a log-transform of the raw count data. Samples Euclidean distance was calculated with the dist function and plotted with pheatmap (v1.0.12) with complete clustering method. Three replicates per timepoint (hiPSC, D10, D20, and D35) and condition (2D and 3D) were used in the RNA-seq analysis for each hiPSC line. Functional annotation of differentially expressed genes was performed on KEGG, Reactome and Gene Ontology databases with ClusterProfiler (v3.16.1)^75^ and ReactomePA (v1.32.0)^76^. Plots summarizing pathway up- and downregulation were generated with ComplexHeatmap v2.16.0^77^. Gene ontology (GO) terms and pathways were reported enriched at a false discovery rate (FDR) of 0.05. Genes were considered differentially expressed for adjusted *p*-value < 0.05 and |log_2_(fold change)| > 1. Differentially expressed genes (DEGs) were intersected with a mitochondrial gene list from MitoCarta 3.0 (Broad Institute) and with genes identified in PD GWAS studies^41^. Gene set enrichment analysis was performed on the Molecular Signatures Database (MSigDB)^30^ C8 cell type signature set (v7.4).

### Reporting summary

Further information on research design is available in the Nature Portfolio Reporting Summary linked to this article.

## Supplementary information

Supplementary Information

Description of Additional Supplementary Files

Supplementary Data 1

Supplementary Data 2

Supplementary Data 3

Supplementary Data 4

Supplementary Data 5

Reporting Summary

## Data Availability

The sequencing data that support the findings of this study have been deposited with the Gene Expression Omnibus (GEO) database and can be accessed through accession number GSE282152. Source data for Fig. 4 are available in Supplementary Data 4, and the Source data for Supplementary Fig. 6 are available in Supplementary Data 5.

## Code availability

Source code for data analysis is available on request.
