# Supplementary material for: Dissecting the transcriptome of Parkin (PRKN)-linked Parkinson’s disease using 2D and 3D hiPSC-neuronal models
Source: Commun Biol. 2026 Jul 22;9:1512. doi: 10.1038/s42003-026-10703-0 (PMC13754778; doi:10.1038/s42003-026-10703-0)
Supplement: Supplementary file 1 [file 42003_2026_10703_MOESM1_ESM.pdf]

Dissecting the transcriptome of Parkin (*PRKN*)-linked Parkinson's disease using 2D and 3D hiPSC-neuronal models

Alessandra Zanon[1,2], Emanuela Kerschbamer[1,3], Diana A. Riekschnitz[1], Valentina Gilmozzi[1], Greta Laurenti[1], Martin Lang[1], Anne Grünewald[4], Philip Seibler[5], Peter P. Pramstaller PP[1], Andrew A. Hicks[1], Christian X. Weichenberger[1], Irene Pichler[1]

[1]*Institute for Biomedicine, Eurac Research, Bolzano, Italy*

[2]*Department of Clinical Pathology, Hospital of Bolzano, Bolzano, Italy*

[3]*Infection Immunology Laboratory, Department of Biomedicine, University of Basel, Basel, Switzerland*

[4]*Luxembourg Centre for Systems Biomedicine (LCSB), University of Luxembourg, Belvaux, Luxembourg*

[5]*Institute of Neurogenetics, University of Lübeck and University Hospital Schleswig-Holstein, Lübeck, Germany*

Address correspondence to: alessandra.zanon@sabes.it

Supplementary Information

Supplementary Table 1. Genotypic and phenotypic information for hiPSC lines included in the present study.

ID	*Mutation*	*Affection status*
PRKN-1 (hiPSC-B125)	*Homozygous PRKN* c.971 del A in ex9, p.Val324AlafsTer111, rs1562519380	affected
PRKN-2 (hiPSC-2011)	*Homozygous PRKN* del ex3	affected
CTRL1 (hiPSC-SFC084-03-02)	/	unaffected
CTRL-2 (hiPSC-802)	/	unaffected

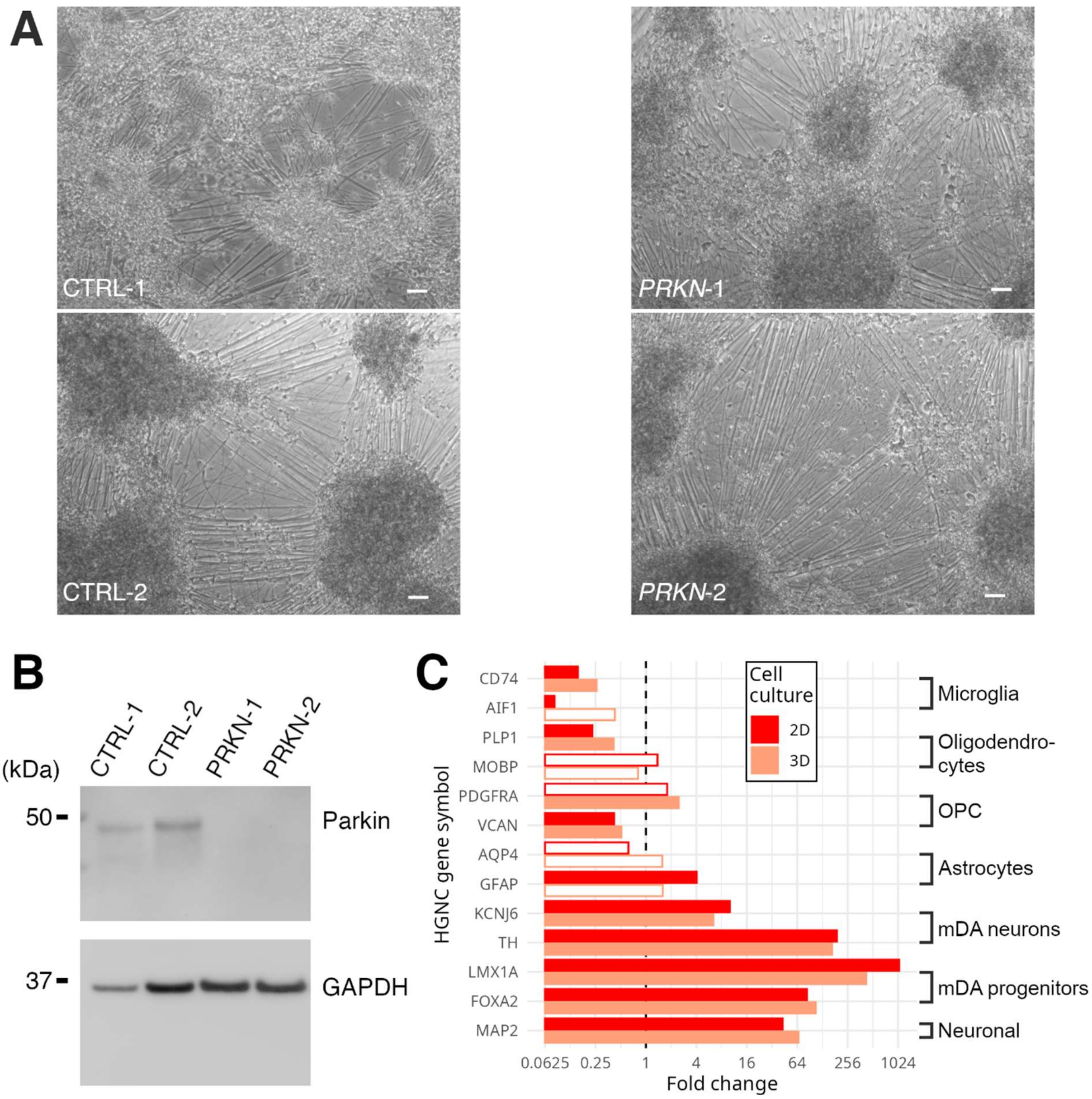
A
CTRL-1
PRKN-1
CTRL-2
PRKN-2
B
CTRL-1
CTRL-2
PRKN-1
PRKN-2
(kDa)
50
37
Parkin
GAPDH
C
CD74
AIF1
PLP1
MOBP
PDGFRA
VCAN
AQP4
GFAP
KCNJ6
TH
LMX1A
FOXA2
MAP2
HGNC gene symbol
Cell culture
2D
3D
Microglia
Oligodendro-cytes
OPC
Astrocytes
mDA neurons
mDA progenitors
Neuronal
0.0625 0.25 1 4 16 64 256 1024
Fold change

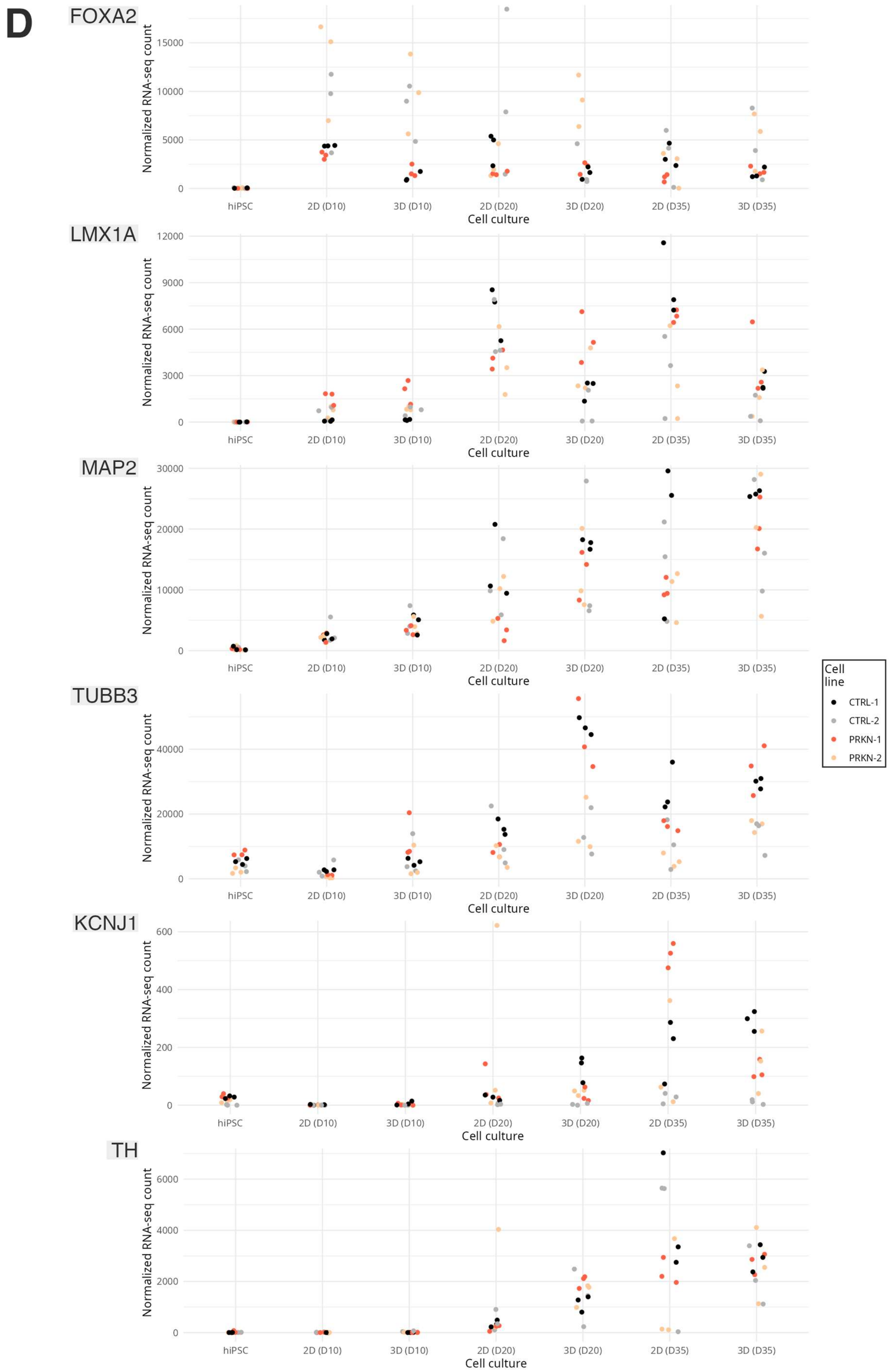

D
FOXA2
LMX1A
MAP2
TUBB3
KCNJ1
TH
Normalized RNA-seq count
Cell culture
hiPSC
2D (D10)
3D (D10)
2D (D20)
3D (D20)
2D (D35)
3D (D35)
Cell line
CTRL-1
CTRL-2
PRKN-1
PRKN-2

Supplementary Figure 1. **Characterization of hiPSC-derived neuronal cultures at day 35 of differentiation.** (**A**) Representative images visualized by phase contrast inverted microscope of hiPSC-derived 2D neurons at day 35 of differentiation. Scale bar=50 µm. (**B**) Western blot analyses showing Parkin protein expression in hiPSC-derived neurons of CTRL-1, CTRL-2, *PRKN*-1, and *PRKN*-2. Parkin levels were analyzed with an anti-Parkin antibody recognizing the C-terminus, GAPDH was used as loading control. Molecular mass markers are in kilodaltons (kDa). n=1 differentiation. (**C**) Differential gene expression of well-established lineage-specific marker genes was analyzed in the RNA-seq datasets of all four lines from both 2D and 3D cultures at day 35 of differentiation: Microglial populations were evaluated using *CD74* and *AIF1* (*IBA1*), mature oligodendrocytes using *PLP1* and *MOBP*, oligodendrocyte progenitor cells (OPCs) using *PDGFRA* and *VCAN*, and astrocytes using *AQP4* and *GFAP*. Neuronal lineage specification was assessed using *FOXA2* and *LMX1A*, which are established ventral midbrain dopaminergic (mDA) patterning markers, while neuronal differentiation and maturation were evaluated using *MAP2* as a pan-neuronal marker. The presence of dopaminergic neurons was further supported by expression of *TH* and *GIRK2* (*KCNJ6*), markers of mature midbrain dopaminergic neurons. Bars represent the expression fold change with respect to differentiation start (hiPSCs) in the RNA-sequencing experiment; the *x*-axis is on a $\log_2$ scale. Values below 1 (dashed vertical line) indicate down-regulation of expression. Red bars display gene expression change in 2D culture, whereas orange bars visualize those from 3D culture. Filled bars indicate statistically significant changes (FDR $\alpha < 0.05$). A single fold change value is derived from three replicates for each comparison presented. (**D**) Plot of RNA-seq count data normalized by estimated size factor (DESeq2 function plotCounts) for the mDA precursor markers *FOXA2* and *LMX1A*, the pan-neuronal markers *MAP2* and *TUBB3* as well as the mDA markers *KCNJ1* and *TH* for both *PRKN*-mutant and CTRL lines in 2D and 3D conditions at hiPSC, day 10, day 20, and day 35, respectively. n=3 independent differentiations.

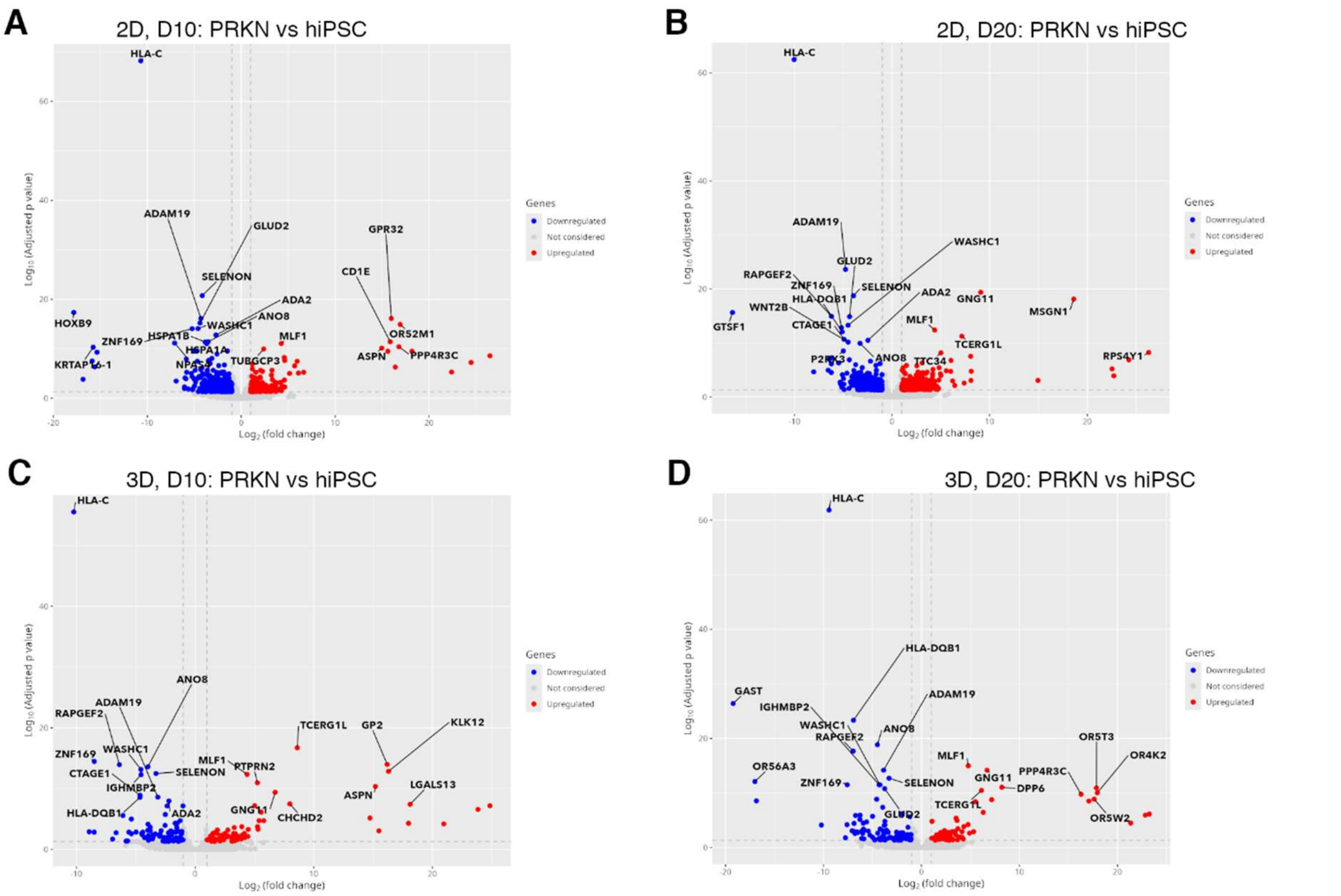

Supplementary Figure 2. **DEGs in *PRKN*-mutant neurons compared to control neurons.** Volcano plots representing $\log_2$(fold change) and $-\log_{10}$(adjusted p value) of genes exclusively altered at D10 (**A**), D20 (**B**), in the 2D culture condition and at D10 (**C**), D20 (**D**) in the Alg/Fn 3D model system, highlighting upregulated genes in red and downregulated genes in blue. n=3 independent differentiations.

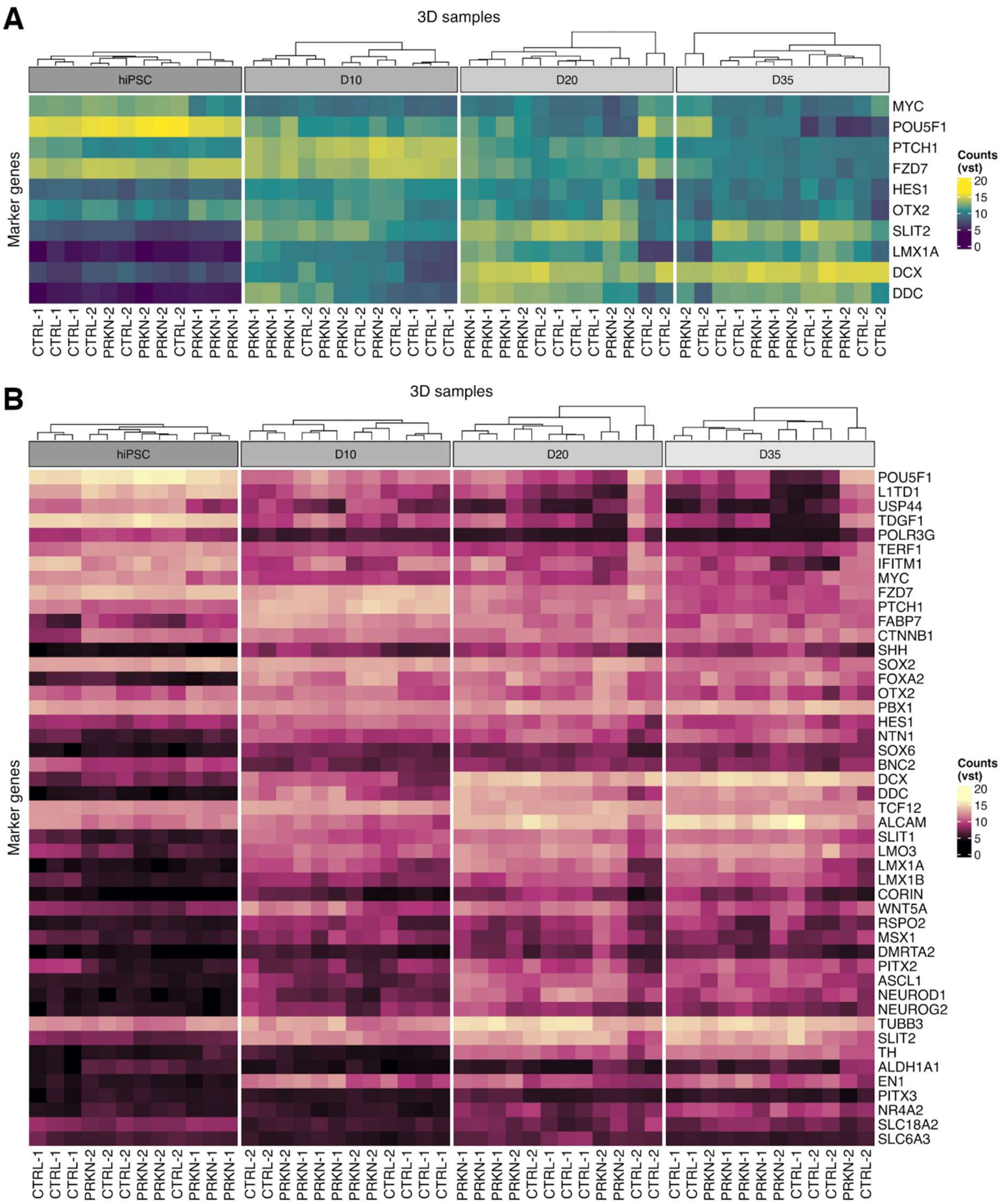

Supplementary Figure 3. **Validation of mDA identity in hiPSC-derived neurons from healthy controls and *PRKN* mutation carriers generated using the 3D culture method.** (**A**) Heatmap showing the transition in gene expression during differentiation from hiPSCs to mDA neurons across D10, D20, and D35 in all four samples of the 3D dataset. Early pluripotency markers (*MYC*, *POU5F1 – OCT4*) progressively decrease, whereas genes associated with mDA specification and differentiation (*PTCH1*, *FZD2*, *HES1*, *OTX2*, *SLIT2*, *LMX1A*) and early mature neuronal markers (*DCX*, *DDC*) increase over time. Each column represents a specific differentiation timepoint for control or *PRKN*-mutant cells, and each row corresponds to the expression of an individual gene. Colors indicate variance-stabilized count data, essentially log_2-transformed raw counts. (**B**) Heatmap showing a more extensive set of marker genes reported in the literature to be relevant and necessary for mDA differentiation, evaluated in the Alg/Fn 3D model system. These markers were selected to recapitulate the *in vivo* developmental trajectory of mDA neurons, spanning multiple transitional stages including hiPSCs, radial glia, neural progenitors, and lineage-committed precursor populations of the developing midbrain. Because several markers are expressed across overlapping developmental stages and cell identities, genes were ordered empirically to reflect progression along the mDA differentiation trajectory rather than grouped into discrete cell type categories. Both panels were adapted from differentiation stage representations described by Novak *et al.*[1]. n=3 independent differentiations.

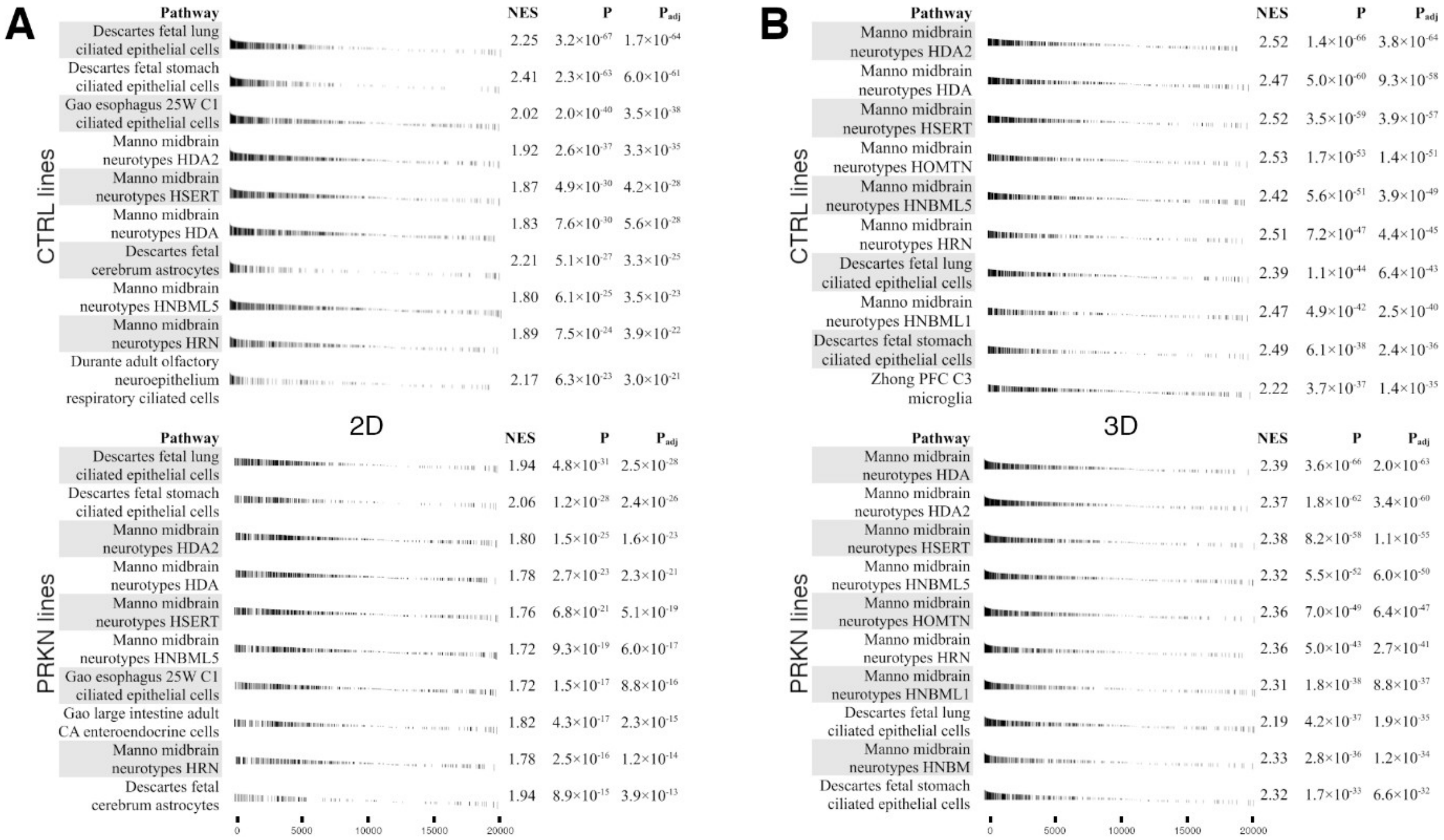

Supplementary Figure 4. **Gene set enrichment analysis of DEGs between hiPSCs and D35 in both control and *PRKN*- mutant cells.** (**A**) Table representing the ten most enriched MSigDB C8 cell type signature gene sets at D35 compared to hiPSC in control (CTRL) and *PRKN*-mutant lines in the 2D culture condition. (**B**) Like panel (A), but for the Alg/Fn 3D model system. Pathway, cell type signature gene set name; NES, Normalized Enrichment Score. n=3 independent differentiations.

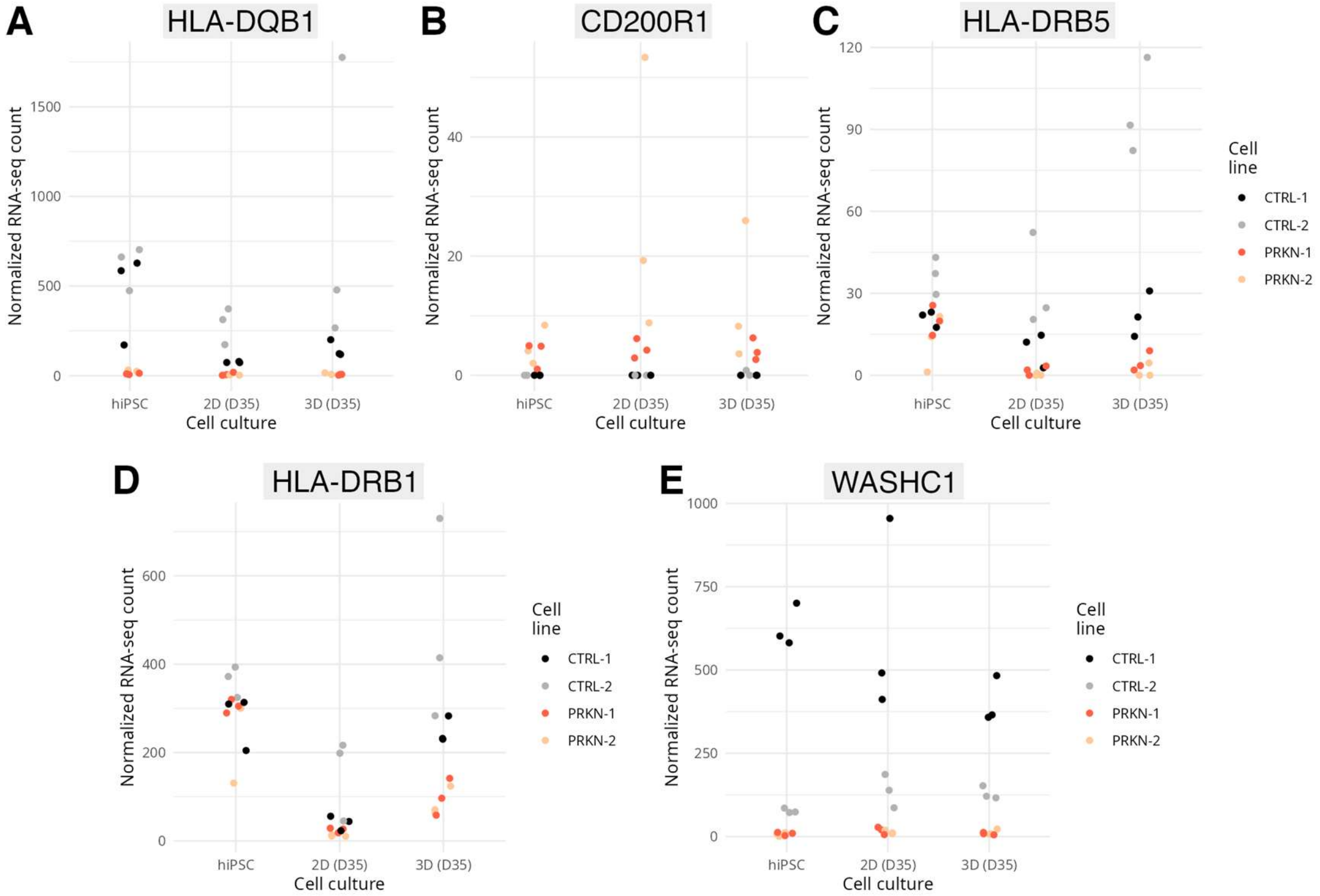

Supplementary Figure 5. RNA-seq expression data for significantly dysregulated genes. Plot of RNA-seq count data normalized by estimated size factor (DESeq2 function plotCounts) for (**A**) *HLA-DQB1*, (**B**) *CD200R1*, (**C**) *HLA-DRB5*, (**D**) *HLA-DRB1*, and (**E**) *WASHC1* for both *PRKN*-mutant and CTRL lines in 2D and 3D conditions (continuation of Figure 5D-F, plotting RNA-seq counts for all genes highlighted in Figure 5A). n=3 independent differentiations.

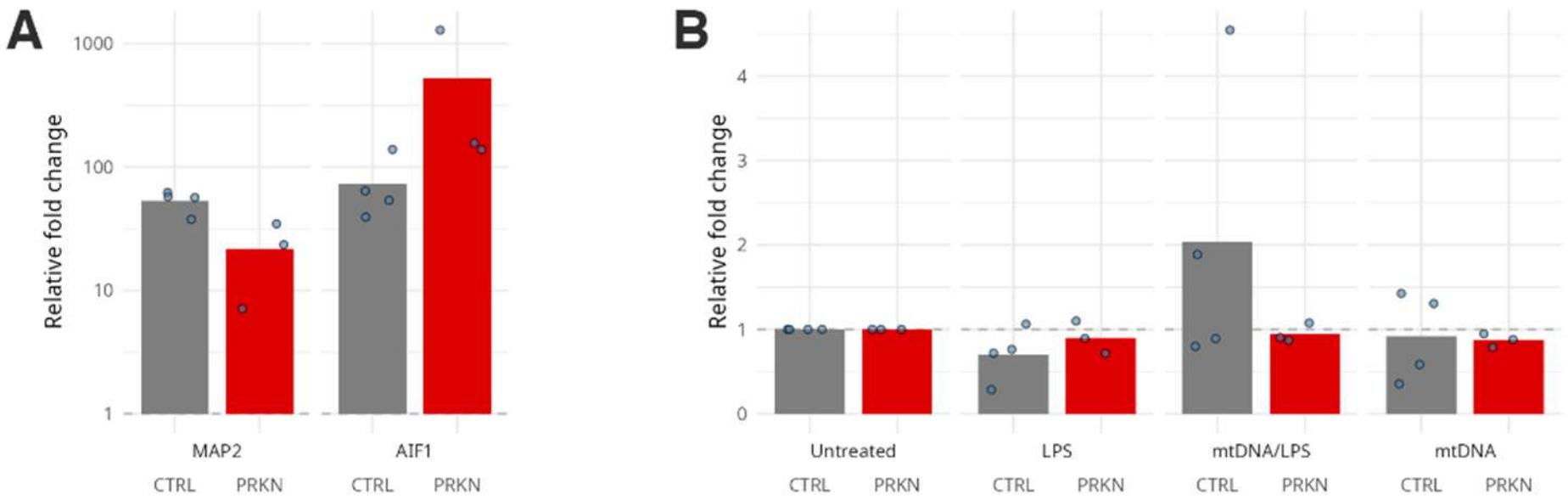

Supplementary Figure 6. qRT-PCR analyses in neuron microglia co-cultures. (**A**) Fold change of the expression of the pan-neuronal marker *MAP2* in untreated neuron-microglia co-cultures compared to undifferentiated hiPSC and the microglial marker *AIF1* (*IBA1*) compared to neurons (n=3 *PRKN*-PD patients, n=4 CTRLs). Figure *y*-axis is on logarithmic scale due to an outlier. (**B**) Fold change of *HLA-C* expression after treatment with lipopolysaccharides (LPS), mitochondrial DNA (mtDNA), and with both (mtDNA/LPS) in neuron-microglia co-cultures (n=3 *PRKN*-PD patients, n=4 CTRLs). Points are single measurements representing $2^{-\Delta\Delta CT}$. n=1 differentiation.

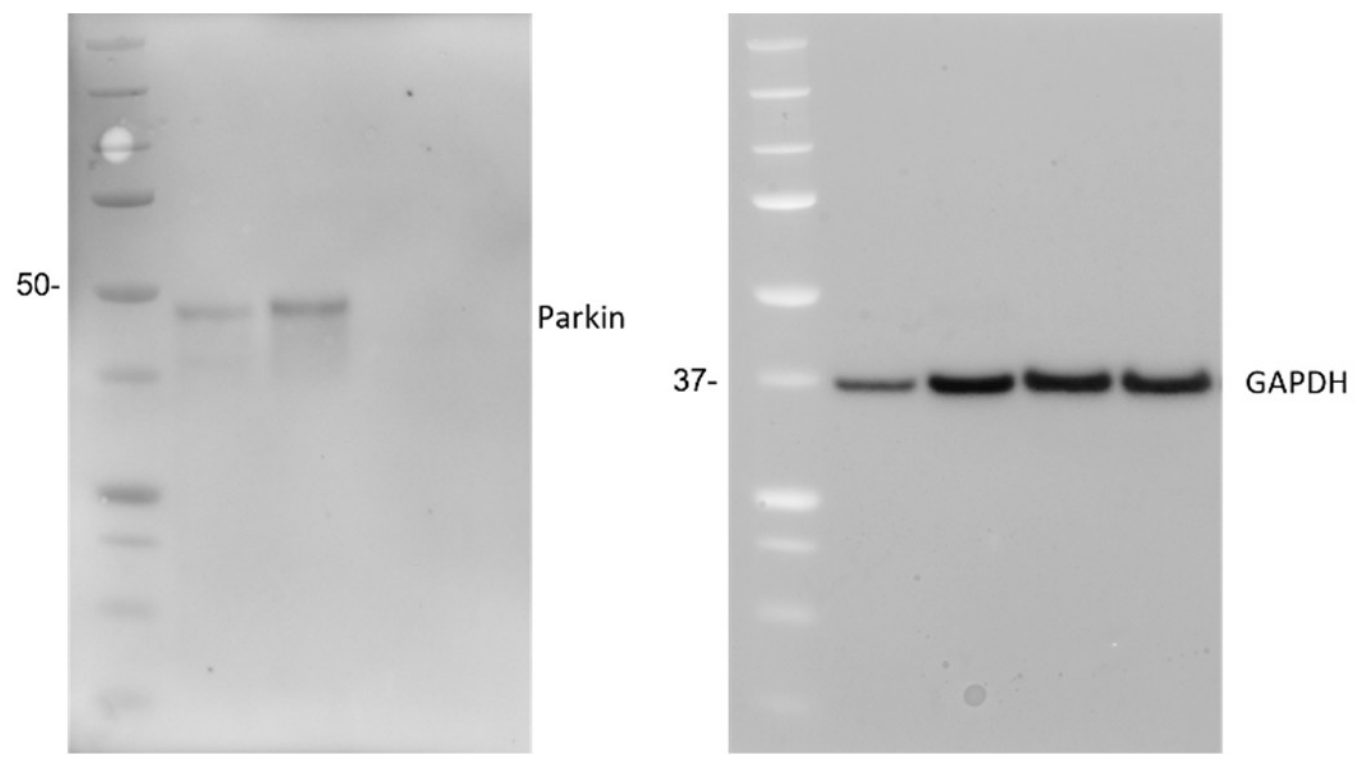

Supplementary Figure 7. Uncropped and unedited Western blot images. Uncropped Western blots of Supplementary Figure 1B.

Supplementary Reference:

1 Novak, G. *et al.* Single-cell transcriptomics of human iPSC differentiation dynamics reveal a core molecular network of Parkinson's disease. *Commun Biol* **5**, 49 (2022). https://doi.org/10.1038/s42003-021-02973-7
