# Supplementary material for: Dissecting the transcriptome of Parkin (PRKN)-linked Parkinson’s disease using 2D and 3D hiPSC-neuronal models
Source: Commun Biol. 2026 Jul 22;9:1512. doi: 10.1038/s42003-026-10703-0 (PMC13754778; doi:10.1038/s42003-026-10703-0)
Supplement: Supplementary file 2 [file 42003_2026_10703_MOESM2_ESM.pdf]

Description of Additional Supplementary Files

File name: Supplementary Data 1

Description: List of DEGs in the PRKN mutant cell lines compared to healthy controls at all timepoints in the 2D and 3D culture conditions.

File name: Supplementary Data 2

Description: List of the DEGs altered in both the 2D and 3D datasets in the PRKN mutants compared to the controls at day 35, subjected to enrichment analysis in KEGG and Reactome pathways as well as Gene Ontology term enrichment.

File name: Supplementary Data 3

Description: List of the downregulated and upregulated genes obtained by intersecting the DEGs in the PRKN mutants compared to the controls at day 35 for the 2D dataset and the Human MitoCarta 3.0 project. List of the downregulated genes obtained by intersecting the DEGs in the PRKN mutants compared to the controls at day 35 for the 3D dataset and the Human MitoCarta 3.0 project.

File name: Supplementary Data 4

Description: Source data for the graphs in Figure 4.

File name: Supplementary Data 5

Description: Source data for the graphs in Supplementary Figure 6.
