# Supplementary material for: Dissecting the transcriptome of Parkin (PRKN)-linked Parkinson’s disease using 2D and 3D hiPSC-neuronal models
Source: Commun Biol. 2026 Jul 22;9:1512. doi: 10.1038/s42003-026-10703-0 (PMC13754778; doi:10.1038/s42003-026-10703-0)
Supplement: Supplementary file 3 [file 42003_2026_10703_MOESM8_ESM.pdf]

nature portfolio

Corresponding author(s): Alessandra Zanon

Last updated by author(s): Jun 12, 2026

Reporting Summary

Nature Portfolio wishes to improve the reproducibility of the work that we publish. This form provides structure for consistency and transparency in reporting. For further information on Nature Portfolio policies, see our Editorial Policies and the Editorial Policy Checklist.

Statistics

For all statistical analyses, confirm that the following items are present in the figure legend, table legend, main text, or Methods section.

n/a	Confirmed	
☐	☒	The exact sample size (n) for each experimental group/condition, given as a discrete number and unit of measurement
☐	☒	A statement on whether measurements were taken from distinct samples or whether the same sample was measured repeatedly
☐	☒	The statistical test(s) used AND whether they are one- or two-sided<br>*Only common tests should be described solely by name; describe more complex techniques in the Methods section.*
☒	☐	A description of all covariates tested
☐	☒	A description of any assumptions or corrections, such as tests of normality and adjustment for multiple comparisons
☐	☒	A full description of the statistical parameters including central tendency (e.g. means) or other basic estimates (e.g. regression coefficient) AND variation (e.g. standard deviation) or associated estimates of uncertainty (e.g. confidence intervals)
☐	☒	For null hypothesis testing, the test statistic (e.g. F, t, r) with confidence intervals, effect sizes, degrees of freedom and P value noted<br>*Give P values as exact values whenever suitable.*
☒	☐	For Bayesian analysis, information on the choice of priors and Markov chain Monte Carlo settings
☒	☐	For hierarchical and complex designs, identification of the appropriate level for tests and full reporting of outcomes
☒	☐	Estimates of effect sizes (e.g. Cohen's d, Pearson's r), indicating how they were calculated

Our web collection on statistics for biologists contains articles on many of the points above.

Software and code

Policy information about availability of computer code

Data collection	All software packages used to generate the RNA-seq data are described in the Materials and Methods section of the manuscript under the subheading "RNA-seq and data analysis".
Data analysis	All software packages used to analyze the RNA-seq data are described in the Materials and Methods section of the manuscript under the subheading "RNA-seq and data analysis".

For manuscripts utilizing custom algorithms or software that are central to the research but not yet described in published literature, software must be made available to editors and reviewers. We strongly encourage code deposition in a community repository (e.g. GitHub). See the Nature Portfolio guidelines for submitting code & software for further information.

Data

Policy information about availability of data

All manuscripts must include a data availability statement. This statement should provide the following information, where applicable:

- Accession codes, unique identifiers, or web links for publicly available datasets
- A description of any restrictions on data availability
- For clinical datasets or third party data, please ensure that the statement adheres to our policy

The sequencing data have been deposited with the Gene Expression Omnibus (GEO) database and can be accessed through accession number GSE282152. To

review GEO accession GSE282152 until the data are published, go to the following link and enter token wpedegycjtqrdgt into the box: https://www.ncbi.nlm.nih.gov/geo/query/acc.cgi?acc=GSE282152

Research involving human participants, their data, or biological material

Policy information about studies with human participants or human data. See also policy information about sex, gender (identity/presentation), and sexual orientation and race, ethnicity and racism.

Reporting on sex and gender	Findings apply to both sexes. Gender was not evaluated. Sex was considered based on self-reporting.
Reporting on race, ethnicity, or other socially relevant groupings	No socially relevant categorization variables were used.
Population characteristics	Sex, age at disease (PD) onset, age at biopsy taking, mutation, zygosity, and affection status of the four individuals, from whom the iPCs were derived, are given in Supplementary Table 1.
Recruitment	We have selected two PD patient lines with different homozygous mutations and two healthy control lines.
Ethics oversight	The study was approved by the ethics committee of the South Tyrolean Health Care System (approval number 102/2014).

Note that full information on the approval of the study protocol must also be provided in the manuscript.

Field-specific reporting

Please select the one below that is the best fit for your research. If you are not sure, read the appropriate sections before making your selection.

☒ Life sciences ☐ Behavioural & social sciences ☐ Ecological, evolutionary & environmental sciences

For a reference copy of the document with all sections, see nature.com/documents/nr-reporting-summary-flat.pdf

Life sciences study design

All studies must disclose on these points even when the disclosure is negative.

Sample size	The sample size (number of cell lines included in the study: 2 patient lines and 2 control lines) is determined by practical feasibility and budget availability.
Data exclusions	No data were excluded.
Replication	We have triplicates in RNA-seq ,and they were used during DESeq2 calculations.
Randomization	Samples were allocated into experimental groups based on their genotype.
Blinding	Investigators were blinded to group allocation during data collection. For the cell culture experiments, cell lines were assigned an ID-code and samples were assigned to genotype groups for data analysis.

Reporting for specific materials, systems and methods

We require information from authors about some types of materials, experimental systems and methods used in many studies. Here, indicate whether each material, system or method listed is relevant to your study. If you are not sure if a list item applies to your research, read the appropriate section before selecting a response.

Materials & experimental systems

n/a	Involved in the study	
☐	☒	Antibodies
☐	☒	Eukaryotic cell lines
☒	☐	Palaeontology and archaeology
☒	☐	Animals and other organisms
☒	☐	Clinical data
☒	☐	Dual use research of concern
☒	☐	Plants

Methods

n/a	Involved in the study	
☒	☐	ChIP-seq
☒	☐	Flow cytometry
☒	☐	MRI-based neuroimaging

Eukaryotic cell lines

Policy information about cell lines and Sex and Gender in Research

Cell line source(s)	Human iPSCs from two control individuals (hiPS-802 - CTRL-2 , female, and hiPS-SFC084-03-02- CTRL-1, female) and two PD patients carrying a biallelic mutation in the PRKN gene (hiPS-B125 - PRKN-1, male, and hiPS-2011, female - PRKN-2) were used. hiPSC-802 and hiPSC-2011 lines were generated in the laboratory of the Institute for Biomedicine . hiPSC-2011: PMID: 31715428. hiPSC line SFC084-03-02 was established through the StemBANCC consortium (https://cells.ebisc.org/STBCi033-B/), hiPSC-B125 was generated at the Institute of Neurogenetics of the University of Lübeck, PMID: 28379402.
Authentication	Described in the respective publications for hiPS-SFC084-03-02, hiPS-B125, hiPS-2011. The line hiPS-802 was authenticated using the same procedure as the other lines including the potential to differentiate into all three germ layers, and a stable karyotype.
Mycoplasma contamination	All four lines tested regularly negative for mycoplasma contamination.
Commonly misidentified lines (See ICLAC register)	None.

Plants

Seed stocks	N/A
Novel plant genotypes	N/A
Authentication	N/A
